# Polydopamine Nanoparticles Targeting Ferroptosis Mitigate Intervertebral Disc Degeneration Via Reactive Oxygen Species Depletion, Iron Ions Chelation, and GPX4 Ubiquitination Suppression

**DOI:** 10.1002/advs.202207216

**Published:** 2023-03-23

**Authors:** Xiao Yang, Yan Chen, Jiadong Guo, Jiaxin Li, Pu Zhang, Huan Yang, Kewei Rong, Tangjun Zhou, Jingke Fu, Jie Zhao

**Affiliations:** ^1^ Shanghai Key Laboratory of Orthopedic Implants Department of Orthopedics Ninth People's Hospital Shanghai Jiao Tong University School of Medicine 639 Zhizaoju Road Shanghai 200011 P. R. China; ^2^ Department of Orthopedics The Second Affiliated Hospital of Harbin Medical University 246 Xuefu Road Harbin 150001 P. R. China; ^3^ The Second Clinical Medical College of Yunnan University of Traditional Chinese Medicine 1076 Yuhua Road Kunming 650500 P. R. China

**Keywords:** ferroptosis, GPX4 ubiquitination, intervertebral disc degeneration, nucleus pulposus, polydopamine nanoparticles

## Abstract

Intervertebral disc degeneration (IVDD)‐induced lower back pain (LBP) is a common problem worldwide. The underlying mechanism is partially accredited to ferroptosis, based on sequencing analyses of IVDD patients from the gene expression omnibus (GEO) databases. In this study, it is shown that polydopamine nanoparticles (PDA NPs) inhibit oxidative stress‐induced ferroptosis in nucleus pulposus (NP) cells in vitro. PDA NPs scavenge reactive oxygen species (ROS), chelate Fe^2+^ to mitigate iron overload, and regulate the expression of iron storage proteins such as ferritin heavy chain (FHC), ferritin, and transferrin receptor (TFR). More importantly, PDA NPs co‐localize with glutathione peroxidase 4 (GPX4) around the mitochondria and suppress ubiquitin‐mediated degradation, which in turn exerts a protective function via the transformation and clearance of phospholipid hydroperoxides. PDA NPs further down‐regulate malondialdehyde (MDA) and lipid peroxide (LPO) production; thus, antagonizing ferroptosis in NP cells. Moreover, PDA NPs effectively rescue puncture‐induced degeneration in vivo by targeting ferroptosis and inhibiting GPX4 ubiquitination, resulting in the upregulation of antioxidant pathways. The findings offer a new tool to explore the underlying mechanisms and a novel treatment strategy for IVDD‐induced LBP.

## Graphical abstract

Polydopamine nanoparticles (PDA NPs) targeting ferroptosis mitigate intervertebral disc degeneration via three main pathways: 1) suppression of the ubiquitylation of GPX4 to maintain its expression and function; 2) chelation of Fe^2+^ to inhibit lipid peroxidation and malondialdehyde (MDA) formation; and (3) recovery of mitochondrial function as a reactive oxygen species (ROS) scavenger.

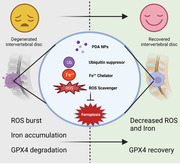

## 1 Introduction

Lower back pain (LBP) caused by intervertebral disc degeneration (IVDD) is the leading cause of disability according to “Global Burden of Disease Study 2019,”^[^ ^1^, ^2^ ^]^ the incidence of which increased by 54% between 1990 and 2015. IVDD affects more than 632 million people globally, resulting in a substantial economic burden.^[^ ^3^, ^4^, ^5^ ^]^ In China, LBP ranks in the top 20 causes of disability‐adjusted life‐years (DALYs), rising from the 17th position in 1990 to the 13th position in 2017. Moreover, LBP is the leading cause of years leading disability (YLDs) burden disease.^[^ ^6^, ^7^ ^]^ Intervertebral discs (IVDs) are flexible joints composed of three parts: the central nucleus pulposus (NP), surrounding annulus fibrosus (AF), and cartilage endplate covering the vertebral bodies. As a connective and compressive forces‐afforded motif,^[^ ^8^, ^9^ ^]^ IVD is prone to degeneration. Inflammatory pathways, such as the activation of the Nuclear Factor Kappa B (NF‐*κ*B) pathway,^[^ ^10^, ^11^ ^]^ the posttranscriptional modification such as N6‐methyladenosine,^[^ ^12^, ^13^ ^]^ and reactive oxygen species (ROS, such as hydrogen peroxide, H_2_O_2_; superoxide anion, O_2_^•−^; and hydroxyl radical, •OH) constitute the harsh oxidative stress microenvironment for the degenerative process.^[^ ^14^, ^15^ ^]^

Apart from these established signaling pathways, recent studies have focused on the role of ferroptosis, an iron‐dependent form of regulated cell death, in IVDD.^[^ ^16^, ^17^ ^]^ Ferroptosis is an iron‐dependent process involved in the downstream signaling pathways of ROS generation.^[^ ^18^ ^]^ The phospholipid peroxidation products (PLOOHs) of ferroptosis can react with both ferrous (Fe^2+^) and ferric (Fe^3+^) ions to produce the PLO• and PLOO• radicals, respectively, further driving the destructive peroxidation chain reaction. Furthermore, along with the changes in the expression of key genes such as glutathione peroxidase 4 (GPX4) and transferrin receptor (TFRC), iron accumulation plays important roles in tumor progression,^[^ ^19^ ^]^ cardiovascular disease,^[^ ^20^ ^]^ and musculoskeletal disorders.^[^ ^21^ ^]^ During human IVDD, ferroptosis acts in a novel chondrocyte subset^[^ ^22^ ^]^ and NP cells, possibly causing neovasculogenesis due to disc herniation following hemoglobin (Hb) accumulation along with heme signal activation.^[^ ^23^ ^]^ Using a puncture‐induced IVDD rat model, previous studies reported that ferroptosis‐targeting therapies via gene‐editing^[^ ^16^, ^24^ ^]^ and homocysteine injection^[^ ^17^ ^]^ were effective in treating IVDD.^[^ ^25^ ^]^ Hu and et al. further demonstrated the efficacy of anti‐ferroptosis therapy in a murine IVDD model using bone mesenchymal stem cell‐secreted extracellular vesicles to inhibit ferroptosis.^[^ ^26^ ^]^ Thus, ferroptosis contributes to IVDD, partially leading to the deterioration of the disc microenvironment. Here, we propose a feasible and novel management technique for IVDD by targeting ferroptosis.

Polydopamine nanoparticles (PDA NPs) are commonly used to scavenge ROS in tumors,^[^ ^27^ ^]^ infections,^[^ ^28^ ^]^ and inflammatory diseases,^[^ ^29^ ^]^ such as periodontal disease.^[^ ^30^ ^]^ Apart from their efficacy in eliminating excessive ROS during inflammation, a recent study revealed the ability of PDA NPs to chelate and absorb Fe ions; thus, exerting an anti‐ferroptosis effect to prevent ischemia/reperfusion injuries in heart disease.^[^ ^31^ ^]^ Moreover, Fe(III)‐chelated PDA NPs inhibit tumor formation.^[^ ^32^ ^]^ We hypothesized that PDA NPs could be effective in treating IVDD‐associated ferroptosis due to their ROS elimination and Fe absorption properties.

In this study, we first reviewed the gene expression omnibus (GEO) database of IVDD to confirm that ferroptosis critically participates in IVDD. We then investigated the feasibility of PDA NPs to inhibit ferroptosis via independent mechanisms, including the canonical mechanism depending on the classical ROS scavenging effect to protect mitochondrial function and Fe^2+^ chelation to prevent the increase of iron ions and ensuing cell homeostasis dysregulation under degeneration. We report the novel use of PDA NPs to suppress the ubiquitin‐mediated degradation of GPX4, a key protein that inhibits ferroptosis in vivo and in vitro. In conclusion, PDA NPs could effectively ameliorate ferroptosis in NP cells in vitro and mitigate the degeneration of the intervertebral discs in vivo.

## 2 Results

### 2.1 Ferroptosis is Involved in IVDD Progression in Humans

**Figure 1:**
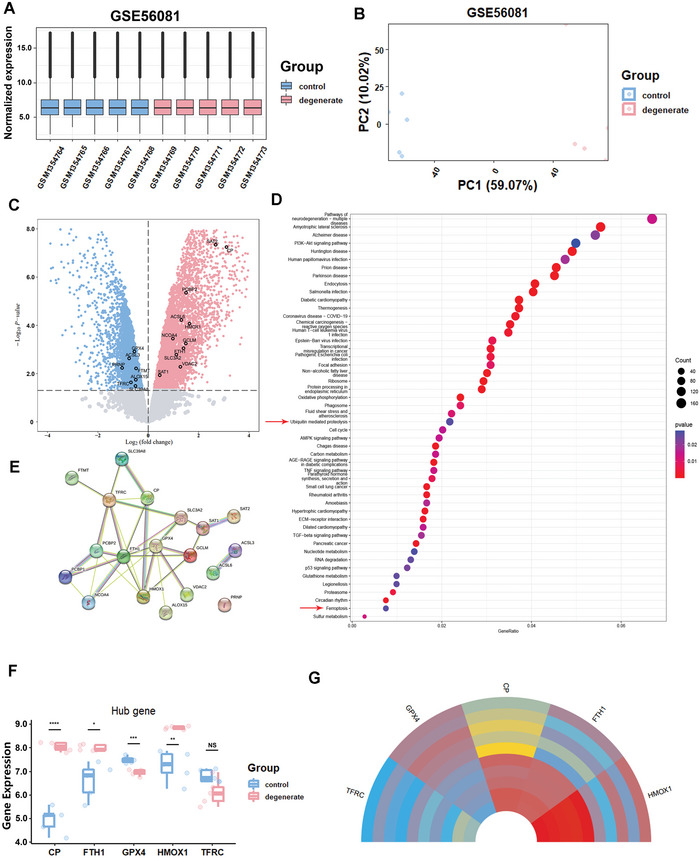
Ferroptosis is involved in the progressive degeneration of intervertebral discs in humans, as determined by the gene expression omnibus (GEO) database. A,B) Normalized expression matrix and principal component analysis (PCA) diagram of GSE56081. C) The volcano plot of GSE56081. D,E) KEGG pathway enrichment analysis and genes associated with ferroptosis enriched by differentially expressed genes (DEGs). F) Validation of hub genes in GSE56081. G) Circos plot for the expression levels of hub genes among different samples. *P < 0.05, **P < 0.01, ***P < 0.001, and ****P < 0.0001.

The GSE56081 expression matrix was normalized, and the distribution trend of boxplots was straight lines (**Figure** **1**A). Principal component analysis (PCA) analysis indicated good repeatability (Figure 1B). After screening with the threshold of *P* value < 0.05, 7859 differentially expressed genes (DEGs) (4364 upregulated and 3495 downregulated) were identified in the GSE56081 dataset. The volcano plot of DEGs is shown in Figure 1C. We identified potential pathways involved in DEGs of IVDD by performing Kyoto Encylopaedia of Genes and Genomes (KEGG) analysis (Figure 1D). Functional analysis demonstrated that among multiple inflammatory and inflammation‐related pathways (Tumor necrosis factor (TNF), Adenosine 5′‐monophosphate (AMP)‐activated protein kinase (AMPK), and transforming growth factor‐*β* (TGF‐*β*) signaling), ubiquitin mediated proteolysis and ferroptosis were involved in IVDD progression. Thus, ferroptosis‐related DEGs were identified. Further analysis revealed five hub genes including ceruloplasmin (CP), ferritin, heavy polypeptide 1 (FTH1), GPX4, heme oxygenase 1 (HOMX1), and TFRC (Figure 1E,F) with the expression levels of these five genes visualized in a circline plot (Figure 1G). Then, the interactive functional plot of these genes was shown in Figure S1A, Supporting Information. For further validation of the involvement of ferroptosis in IVDD, GSE27494 was also included and the results of KEGG pathway enrichment were consistent with the GSE56081 dataset analysis (Figure S1B–E, Supporting Information). Moreover, immunohistochemistry (IHC) revealed that the protein expression levels of GPX4 decreased and ubiquitin significantly increased in human IVDD samples (Figure S1F,G, Supporting Information).

### 2.2 Characterization of PDA NPs

**Figure 2:**
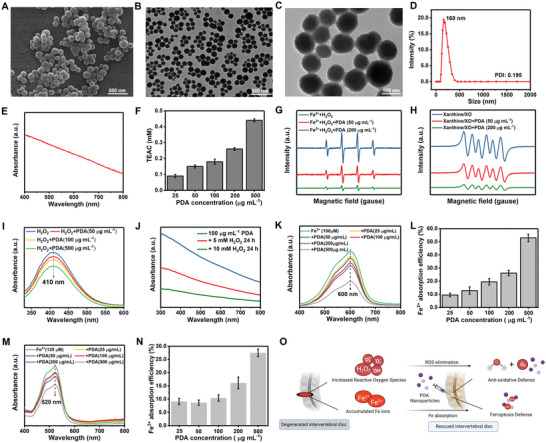
Characterization of PDA NPs. A) Scanning electron microscopy (SEM) image of PDA NPs. B,C) Transmission electron microscopy (TEM) images of PDA NPs. D) The average hydrodynamic particle diameter of PDA NPs. E) Ultraviolet–visible (UV–vis) absorption spectroscopy of PDA NPs. F) Antioxidant capacity of PDA NPs measured by the 2,2″‐azino‐bis (3‐ethylbenzothiazoline‐6‐sulfonic acid) (ABTS) method. G,H) Electron spin resonance (ESR) spectra of DMPO/•OH adducts (G) and DMPO/O_2_•− (H), indicating the depletion of •OH and O_2_•− upon incubation with PDA NPs. I) UV–vis absorption spectra of peroxide–titanium after the addition of PDA NPs, indicating the H_2_O_2_ depletion of PDA NPs (titanium sulfate method). J) UV–vis absorption spectra and PDA NPs after reaction with H_2_O_2_, indicating the degradation of PDA NPs. K) UV–vis absorption spectra of blue tripyridyltriazine‐Fe^2+^ complex after the addition of PDA NPs, indicating the Fe^2+^‐chelating capacity of PDA NPs. L) Fe^2+^ ions chelation efficiency of PDA NPs. M) UV–vis absorption spectra of pink 2,2″‐bipyridine‐Fe^3+^ complex after the addition of PDA NPs, indicating the Fe^3+^‐chelating capacity of PDA NPs. N) Fe^3+^ ions chelation efficiency of PDA NPs. O) Illustration of the reactive oxygen species (ROS) depletion and Fe ions chelation activities of PDA NPs, which are expected to facilitate the anti‐oxidative and ferroptosis defense microenvironment and rescue the degenerated intervertebral disc.

PDA NPs were prepared via the classical Stöber method with some modifications.^[^ ^34^ ^]^ The prepared PDA NPs were uniform spheres with good dispersity, as indicated by the scanning electron microscopy (SEM) and transmission electron microscopy (TEM) images (**Figure** **2**A–C). The average hydrodynamic particle diameter of the PDA NPs was ≈160 nm with a narrow size distribution, further suggesting good dispersion of the NPs in water (Figure 2D). The ultraviolet–visible (UV–vis) absorption spectrum of PDA NPs revealed absorbance in the wide range of 400–800 nm (Figure 2E). The antioxidant capability of PDA NPs was initially investigated using the well‐established 2,2'‐azino‐bis (3‐ethylbenzthiazoline‐6‐sulfonic acid (ABTS) method. ABTS can be oxidized by ROS to green ABTS^•+^, which can be suppressed in the presence of antioxidants. The evaluation of the ABTS^•+^ level; thus, represents the Trolox equivalent antioxidant capacity (TEAC) of the analyte, which revealed that the TEAC value was positively correlated with the concentration of PDA NPs (Figure 2F), suggesting the antioxidant activity of PDA NPs. The antioxidant capacity was further verified by exploring the ROS (•OH and O_2_^•−^) depleting ability of PDA NPs by electron spin resonance (ESR) using DMPO (5,5‐dimethyl‐1‐pyrroline N‐oxide) as the spin probe. As displayed in Figure 2G, the characteristic DMPO/•OH signal showed a concentration‐dependent decline after treatment with PDA NPs, elucidating the •OH scavenging capacity of PDA NPs. Similarly, the characteristic signal intensity of DMPO/O_2_^•−^ showed a distinct decline upon PDA NPs addition, which could be further decreased by promoting the concentration of the NPs (Figure 2H). This result demonstrated the excellent O_2_^•−^ scavenging activity of PDA NPs. The H_2_O_2_ scavenging capacity of PDA NPs was then evaluated using the titanium sulfate method. Titanium sulfate can react with H_2_O_2_ to spectrophotometrically produce a peroxide–titanium complex, which can be suppressed in the presence of antioxidants. As shown in Figure 2I, the characteristic peak of peroxide‐titanium at 410 nm decreased in a dose‐dependent manner upon PDA NPs addition, demonstrating the concentration‐dependent H_2_O_2_ depletion of PDA NPs. Along with H_2_O_2_ depletion, the concentration of PDA also declined after co‐incubation with H_2_O_2_ for 24 h, suggesting the biodegradability of PDA NPs in the presence of ROS (Figure 2J).

Considering the central role of iron in ferroptosis, iron chelation may potentially regulate the iron‐dependent ferroptotic cell death in disc disorders. Accordingly, we tested the Fe^2+^ and Fe^3+^ ions chelating capacity of PDA NPs. Fe^2+^ ions can react with tripyridyltriazine to form a blue tripyridyltriazine‐Fe^2+^ compound. The Fe^2+^ ions chelating capacity of PDA NPs was thus evaluated by measuring the tripyridyltriazine‐Fe^2+^ level. As shown in Figure 2K,L, the characteristic peak of tripyridyltriazine‐Fe^2+^ at 600 nm displays a concentration‐dependent decrease in the presence of PDA NPs, demonstrating the effective Fe^2+^ ions chelating activity of PDA NPs. Fe^3+^ ions can react with 2,2″‐bipyridine to generate a pink 2,2″‐bipyridine‐Fe^3+^ compound. Correspondingly, the Fe^3+^ ion chelating capacity was investigated by assessing the 2,2″‐bipyridine‐Fe^3+^ level following the addition of PDA NPs. Upon PDA NPs addition, a concentration‐dependent decrease of characteristic 2,2″‐bipyridine‐Fe^3+^ absorbance at 520 nm was observed (Figure 2M,N), confirming the Fe^3+^ ion chelating activity of PDA NPs. Overall, the ROS scavenging capacity and iron chelating activity of PDA NPs are expected to elicit a defense mechanism against cytotoxic redox‐active iron and oxidative stress, showing potential for IVDD treatment (Figure 2O).

### 2.3 PDA NPs Scavenge ROS and Chelate Fe^2+^ to Mitigate the Ferroptosis of NP Cells In Vitro

**Figure 3:**
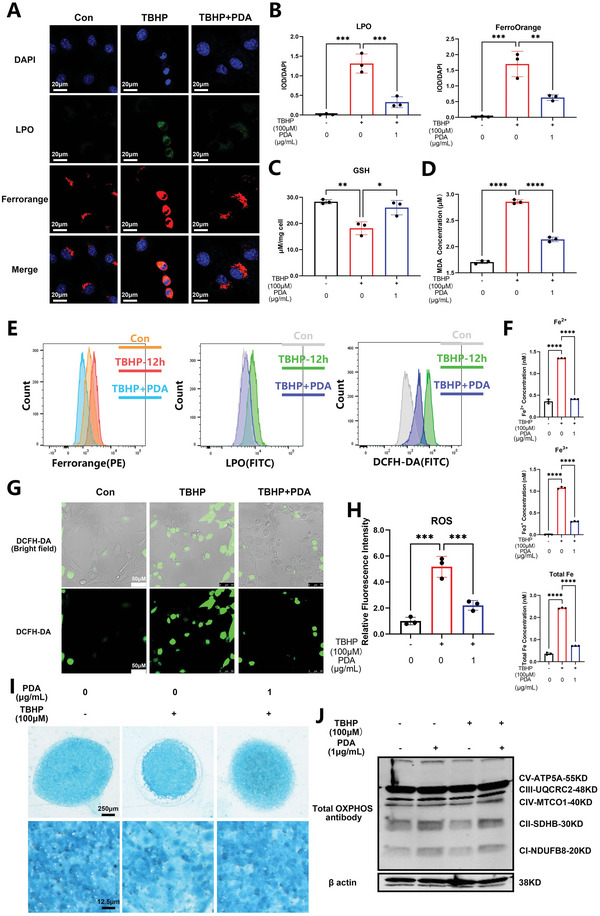
PDA NPs scavenge reactive oxygen species (ROS) and chelate Fe^2+^ ions to mitigate the ferroptosis of nucleus pulposus (NP) cells in vitro. A) Immunofluorescence analysis of lipid peroxide (LPO) and FerroOrange of NP cells stimulated with tert‐butyl hydroperoxide (TBHP) (100 µm) for 12 h and/or pretreated with PDA NPs (1 µg mL^−1^) for 24 h. B) Quantification of integrated optical density (IOD)/4′,6‐diamidino‐2‐phenylindole (DAPI) of the LPO and FerroOrange. C) GSH assay of NP cells stimulated with TBHP (100 µm) for 12 h and/or pretreated with PDA NPs (1 µg mL^−1^) for 24 h. D) Malondialdehyde (MDA) quantification of NP cells stimulated with TBHP (100 µm) for 12 h and/or pretreated with PDA NPs (1 µg mL^−1^) for 24 h. E) Flowcytometry analysis of PE‐FerroOrange, FITC‐LPO, and FITC‐ROS in NP cells stimulated with TBHP (100 µm) for 12 h and/or pretreated with PDA NPs (1 µg mL^−1^) for 24 h. F) Quantification of the iron amount (Fe^2+^, Fe^3+^) in NP cells stimulated with TBHP (100 µm) for 12 h and/or pretreated with PDA NPs (1 µg mL^−1^) for 24 h. G) Immunofluorescence analysis of ROS (DCFH‐DA) in NP cells stimulated with TBHP (100 µm) for 12 h and/or pretreated with PDA NPs (1 µg mL^−1^) for 24 h. H) Quantification of relative fluorescence intensity of the NP cells. I) Alcian blue stain of the high‐density culture of NP cells stimulated with TBHP (100 µm) for 12 h and/or pretreated with PDA NPs (1 µg mL^−1^) for 24 h. J) Western blot analysis of total oxidative phosphorylation (OXPHOS) in NP cells stimulated with PDA NPs alone for 24 h, TBHP (100 µm) for 12 h, and/or pretreated with PDA NPs (1 µg mL^−1^) for 24 h. All data are presented as mean ± SD from three replicates. *P < 0.05, **P < 0.01, ***P < 0.001, and ****P < 0.0001.

CCK8 and cytotoxicity tests revealed that the safe concentration of PDA NPs was between 0 to 1 µg mL^−1^, in a period of 24 to 72 h (with an IC_50_ of 3.748 µg mL^−1^; Figure S2A,B, Supporting Information). Using t‐butylhydroperoxide (TBHP) to trigger the ferroptosis of NP cells as previously described, we confirmed that PDA NPs (1 µg mL^−1^) efficiently and significantly suppressed cell death using Dead/Live cell staining (Figure S4D, Supporting Information) with a lower propidium Iodide (PI)/Calcein rate in TBHP+PDA NPs group compared with the TBHP group (Figure S4F, Supporting Information), along with decreased lipid peroxide (LPO) production and Fe^2+^ accumulation (**Figure** **3**A,B), based on the LPO and FerroOrange assay. This was followed by an inhibitory effect on the malondialdehyde (MDA) production induced by TBHP (Figure 3D). The flowcytometry assay of LPO and FerroOrange revealed similar results (Figure 3E). The underlying mechanism could involve the scavenging of oxidative stress by PDA NPs based on their canonical function revealed by flowcytometry (Figure 3E) and Dichlorodihydrofluorescein diacetate (DCFH‐DA) probe (Figure 3G,H). PDA NPs could chelate Fe^2+^; thus, inhibiting the up‐regulation of Fe^2+^, Fe^3+^, and total Fe induced by TBHP (Figure 3F). Finally, PDA NPs administration also rescued glutathione (GSH) production (Figure 3C), which might be regulated via glutathione peroxidases (GPXs) enzymes. To inhibit NP cell ferroptosis, we observed that the catabolic status caused by TBHP was significantly reduced after PDA NPs administration (Figure 3I; Figure S3A, Supporting Information). Considering that mitochondrial dysregulation is one of the most important phenomena of ferroptosis, we monitored the oxidative respiratory chain using western blotting and found that PDA NPs administration enhanced the oxidative phosphorylation (OXPHOS) antibody function and rescued the decreased OXPHOS induced by TBHP (Figure 3J; Figure S3B,C, Supporting Information).

### 2.4 PDA NPs Inhibit the Ubiquitination of GPX4 to Suppress the Ferroptosis of NP Cells In Vitro

**Figure 4:**
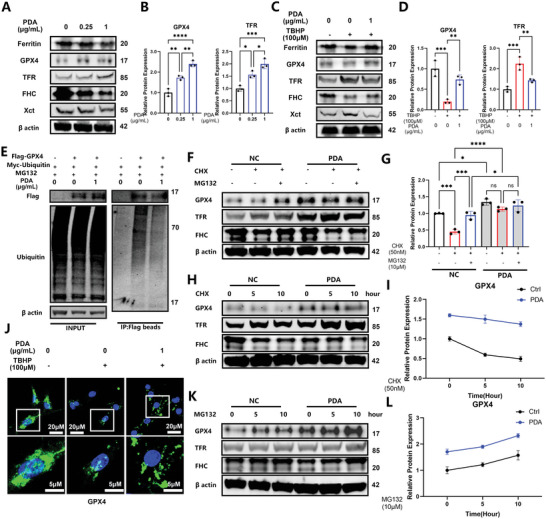
PDA NPs inhibit the ubiquitination of GPX4 to suppress the ferroptosis of nucleus pulposus (NP) cells in vitro. A) Western blot analysis of ferritin, GPX4, TFR, FHC, and Xct in NP cells treated with different concentrations of PDA NPs (0.25, 1 µg mL^−1^) for 24 h. B) Relative protein quantification of grey scale value for GPX4 and TFR. C) Western blot analysis of ferritin, GPX4, TFR, FHC, and Xct in NP cells stimulated with tert‐butyl hydroperoxide (TBHP) (100 µm) for 12 h and/or pretreated with PDA NPs (1 µg mL^−1^) for 24 h. D) Relative protein quantification of grey scale value for GPX4 and TFR. E) Ubiquitylation analysis of GPX4 in 293T cells treated with MG132 (10 µm) and/or PDA NPs (1 µg mL^−1^) using Flag‐GPX4 and Myc‐ubiquitin plasmids with Flag‐tagged beads. F) Western blot analysis of GPX4, TFR, and FHC using *β*‐actin as the loading control in NP cells (pretreated with or without PDA NPs) treated with cycloheximide (50 nm) and MG132 (10 µm). G) Relative protein quantification of grey scale value for GPX4. H) Western blot analysis of GPX4, TFR, and FHC using *β*‐actin as the loading control in NP cells (pretreated with or without PDA NPs) treated with cycloheximide (50 nm) for 0, 5, and 10 h. I) Relative protein quantification of grey scale value for GPX4. J) Immunofluorescence analysis of GPX4 in NP cells stimulated with TBHP (100 µm) for 12 h and/or pretreated with PDA NPs (1 µg mL^−1^) for 24 h. K) Western blot analysis of GPX4, TFR, and FHC using *β*‐actin as the loading control in NP cells (pretreated with or without PDA NPs) treated with MG132 (10 µm) for 0, 5, and 10 h. L) Relative protein quantification of grey scale value for GPX4. All data are presented as mean ± SD from three replicates. *P < 0.05, **P < 0.01, ***P < 0.001, and ****P < 0.0001.

KEGG analysis suggested that ubiquitin mediated proteolysis was involved in IVDD (Figure 1D). Therefore, we further investigated the molecular mechanisms underlying the inhibition of ferroptosis by PDA NPs using increasing concentrations of PDA NPs (0.25 and 1 µg mL^−1^). Consequently, we observed that the Ferritin, ferritin heavy chain (FHC), and Xct expression were down‐regulated (which might be the consequence of Fe^2+^ chelator), but GPX4 and TFR production was significantly up‐regulated (**Figure** **4**A,B). Using TBHP as a ferroptosis trigger, we observed that PDA NPs rescued ferroptosis by down‐regulating the increased protein level of TFR and Xct, and recovering the decreased protein levels of GPX4, Ferritin, and FHC (Figure 4C,D), consistent with the mRNA levels of Fth1, Tfrc, and Slc7a11 (Figure S3E, Supporting Information). However, we did not observe a similar effect on the mRNA and protein levels of GPX4 (Figures S2C and S3E, Supporting Information), indicating the possibility of post‐translational modification in GPX4 expression. Immunofluorescence revealed that PDA NPs efficiently recovered the expression of GPX4 down‐regulated by TBHP (Figure 4J; Figure S4B, Supporting Information). GPX4 is degraded via the ubiquitin‐mediated proteasome pathway; thus, we constructed Flag‐tagged GPX4 and Myc‐tagged Ubiquitin for subsequent degradation assays. Using 293T cells for transfection, we found that GPX4 was poly‐ubiquitinated and PDA NPs administration effectively inhibited this ubiquitination (Figure 4E). Similarly, PDA NPs inhibited the ubiquitination of GPX4 in NP cells (Figure S4E, Supporting Information). To further confirm this finding, we used cycloheximide (a protein synthesis inhibitor, CHX, 50 nm) and MG132 (a proteasome inhibitor, 10 µm) to explore the degradation of GPX4. Using CHX to inhibit the synthesis of GPX4 in the CHX group and using MG132 to inhibit the ubiquitin mediated protein degradation in the CHX+MG132 group, we found that the protein level of GPX4 was recovered, similar to the expression level in the CHX + PDA NPs group, indicating that PDA NPs could effectively antagonize the degradation of GPX4 (Figure 4F,G). In contrast, TFR expression was unchanged during this process and FHC was not rescued with PDA NPs administration (Figure 4F,G). GPX4 was subsequently degraded with the use of CHX for 0, 4, and 8 h, but PDA NPs inhibited the degradation (Figure 4H,I). The protein level of GPX4 was increased, especially in the PDA NPs group after MG132 treatment (Figure 4K,L; Figure S5A, Supporting Information). Interestingly, we found that PDA NPs could not only increase the mRNA transcription of Tfrc but they also inhibit the ubiquitination of TFR simultaneously (Figures S2C and S5B, Supporting Information).

### 2.5 PDA NPs Are Absorbed Via Endocytosis and Co‐Localize With Mitochondria and GPX4 in NP Cells In Vitro

**Figure 5:**
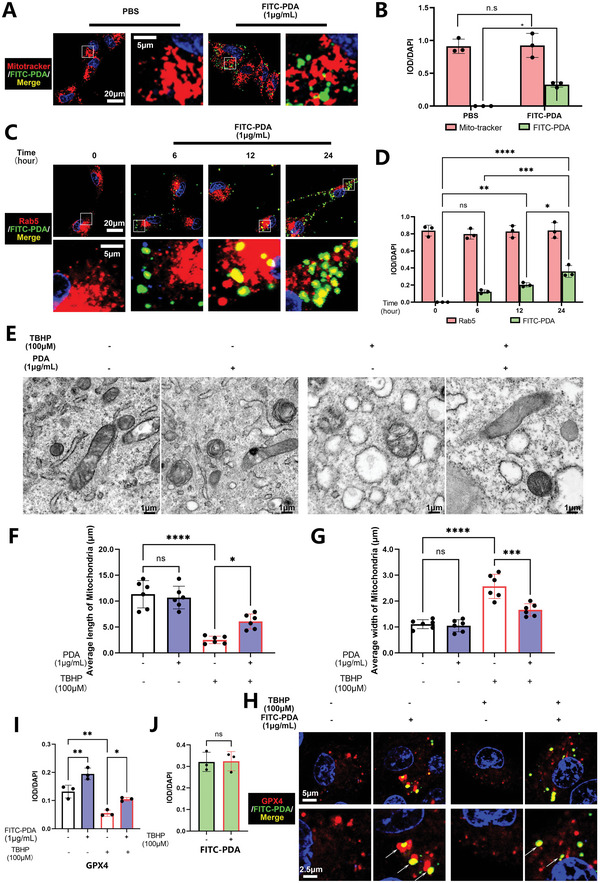
PDA NPs were absorbed via endocytosis and co‐localized with the mitochondria and GPX4 in NP cells in vitro. A) Immunofluorescence analysis of Mito‐tracker and FITC‐PDA NPs in NP cells treated with or without PDA NPs (1 µg mL^−1^) for 24 h. B) Quantification of integrated optical density (IOD)/4′,6‐diamidino‐2‐phenylindole (DAPI) for the Mito‐tracker and FITC‐PDA NPs. C) Immunofluorescence analysis of Rab5 and FITC‐PDA NPs in NP cells treated with PDA NPs (1 µg mL^−1^) for 0, 6, 12, and 24 h. D) Quantification of IOD/DAPI for the Rab5 and FITC‐PDA NPs. E) Transmission electron microscopy (TEM) analysis of NP cells stimulated with PDA NPs alone for 24 h, TBHP (100 µm) for 12 h, and/or pretreated with PDA NPs (1 µg mL^−1^) for 24 h. F,G) Quantification of average length and width of mitochondria. H) Immunofluorescence analysis of GPX4 and FITC‐PDA NPs in NP cells stimulated with FITC‐PDA NPs alone for 24 h, TBHP (100 µm) for 12 h, and/or pretreated with FITC‐PDA NPs (1 µg mL^−1^) for 24 h. I) Quantification of IOD/DAPI for the GPX4. J) Quantification of IOD/DAPI for the FITC‐PDA NPs. All data are presented as mean ± SD from three or six replicates. *P < 0.05, **P < 0.01, ***P < 0.001, and ****P < 0.0001.

Next, we constructed Fluorescein Isothiocyanate (FITC) labeled PDA NPs to investigate the mechanism of PDA NPs entering the cell cytoplasm and tracked its distribution in NP cells. Using immunofluorescence, we found that the FITC‐PDA NPs were spread around the cytoplasm and mainly co‐localized with mitochondria in NP cells after 24 h (**Figure** **5**A,B). With the increase in treatment time of FITC‐PDA NPs from 0 to 24 h, we found that the intensity of FITC‐PDA NPs was higher and the FITC‐PDA NPs were co‐localized with the endocytosis marker Rab5 (Figure 5C,D). Moreover, pharmacological inhibition of endocytosis via Pistop2^[^ ^35^ ^]^ and Hydroxy Dynasore^[^ ^36^ ^]^ decreased the intensity of FITC‐PDA NPs (Figure S6B,C, Supporting Information), indicating that the FITC‐PDA NPs were absorbed into the cell via endocytosis. After being absorbed into cells, the FITC‐PDA NPs gathered around the mitochondria to exert protective functions (Figure S6A, Supporting Information). Using TEM, we found that TBHP stimulation resulted in the expansion of mitochondria in NP cells and obscuring of the mitochondrial cristae, which is a typical marker of ferroptotic mitochondria, with decreased length and increased width^[^ ^31^ ^]^ (Figure 5E). Contrarily, PDA NPs exerted protective effects on mitochondria (Figure 5E), with increased average length and decreased average width of mitochondria (Figure 5F,G) in the TEM test. Moreover, we used the Seahorse test to analyze the oxygen consumption rate (OCR, mitochondrial respiration) and extracellular acidification rate (ECAR, glycolytic function) in NP cells. We observed that PDA NPs antagonized the detrimental effects of TBHP, and recovered the basal and maximal respiration, but did not spare respiratory capacity in the OCR assay (Figure S7A,B, Supporting Information). For ECAR assay, PDA NPs mitigated the increased glycolytic capacity, glycolysis, and glycolytic reserve (Figure S7C,D, Supporting Information). Furthermore, we observed that the FITC‐PDA NPs co‐localized with GPX4 and inhibited its ubiquitin‐mediated degradation, resulting in the up‐regulation of its protein expression (Figure 5H,I). The intensity of FITC‐PDA NPs remained stable after TBHP treatment (Figure 5H,J).

### 2.6 PDA NPs Reduce Ferroptosis, Mitigate IVDD in Rats, and Target GPX4 Ubiquitination In Vivo

**Figure 6:**
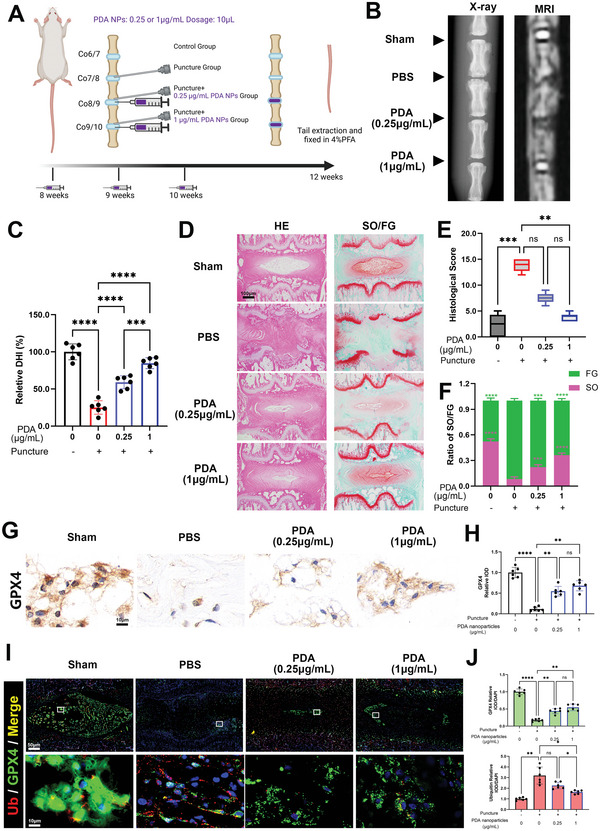
PDA NPs reduce ferroptosis and mitigate IVDD in rats by targeting GPX4 ubiquitination in vivo. All rats (*n* = 6) were punctured at Co7/8, Co8/9, and Co9/10; then, Co8/9 and Co9/10 were rescued by PDA NPs in a concentration of 0.25 and 1 µg mL^−1^ (Sham group: Co6/7, PBS group: Co7/8, PDA Low concentration group: Co8/9, PDA high concentration group: Co9/10). A) Puncture induced IVDD model in rats and relative PDA NPs treatment methods. B) X‐ray and MRI of rat tails focused on the area of operation. C) Quantification of relative DHI in the sections. D) Safranin O‐Fast Green and hematoxylin and eosin (H&E) staining of paraffin sections of the rat tails. E) Quantification of histological score in the sections. F) Quantification of IOD ratio of SO/FG stain of the sections. G) Immunohistochemistry analysis of paraffin sections of the rat tails. H) Quantification of the relative IOD of GPX4. I) Immunofluorescence analysis of paraffin sections of the rat tails. J) Quantification of relative IOD/DAPI of GPX4 and ubiquitin in the sections. All data are presented as mean ± SD from six replicates. *P < 0.05, **P < 0.01, ***P < 0.001, and ****P < 0.0001.

To further establish the efficacy of PDA NPs, we constructed a canonical puncture‐induced degeneration model in rats as previously described.^[^ ^37^, ^38^ ^]^ We used an injection interval of once per week according to a previous study^[^ ^30^ ^]^ (**Figure** **6**A) and our observation was that PDA NPs were degraded by half after 48 h and by almost 90% between 7 to 14 days (Figure S7E,F, Supporting Information). Moreover, two concentrations of PDA NPs (0.25 and 1 µg mL^−1^) could effectively mitigate the degeneration of intervertebral discs. The main manifestations were the recovery of lost disc height and disc intensity (Figure 6B). We observed that the decreased relative disc height index (DHI) induced by puncture was significantly increased with PDA NPs administration (Figure 6C). Using histological sections to further evaluate the histological change, we found that the discs of Co7/8 suffered serious degeneration with a puncture, which displayed loss of nucleus pulposus tissue, fibrosis of the disc, and even destruction to the cartilage in the growth plate (Figure 6D). PDA NPs administration decreased the histological score (to evaluate the degree of degeneration; the higher the scores, the more severe the degeneration) (Figure 6D,E). Moreover, the Safranin O‐Fast Green (SO/FG) staining test revealed that the PDA NPs inhibited the fibrosis formation (FG staining) and loss of nucleus pulposus tissues (SO staining) in discs, which displayed as the recovery of the ratio of SO/FG stain in the IVD area (Figure 6D,F). Immunohistochemistry revealed that the expression of GPX4 decreased after a puncture, but PDA NPs administration recovered its expression and the relative integrated optical density (IOD) level was significantly increased (Figure 6G,H). Moreover, using immunofluorescence to double label GPX4 and ubiquitin in discs, we found that the intensity of ubiquitin was increased and the expression of GPX4 was decreased in the puncture‐induced degeneration group (Figure 6I,J), leading to further ferroptosis and disc degeneration. However, PDA NPs reduced the expression of ubiquitin and recovered the intensity of GPX4 in the disc area (Figure 6I,J), which suppressed ferroptosis and mitigated the degeneration of IVDs. To further confirm this result, we isolated the protein within rat tail NP and evaluated the ubiquitination of GPX4 by co‐immunoprecipitation (Figure S9A, Supporting Information). We observed that GPX4 levels were decreased and ubiquitin was increased in the puncture group, but in the PDA NPs treated groups (0.25 and 1 µg mL^−1^) the GPX4 levels were recovered and ubiquitin was slightly decreased. Thus, PDA NPs suppressed GPX4 ubiquitination in our in vivo ubiquitylation assay (Figure S9B, Supporting Information).

## 3 Discussion

**Figure 7:**
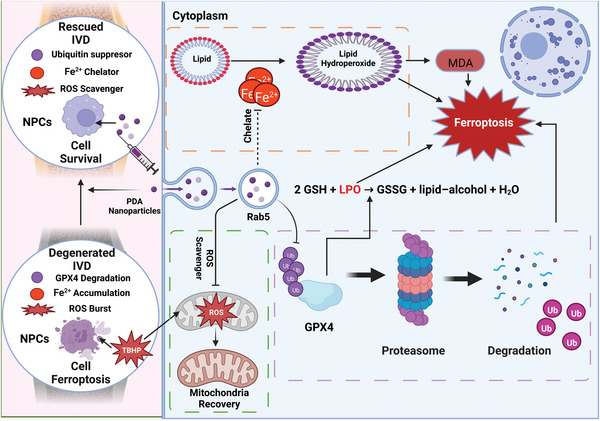
PDA NPs targeting ferroptosis mitigate intervertebral disc degeneration via three main pathways: 1) suppression of the ubiquitylation of GPX4 to maintain its expression and function; 2) chelation of Fe^2+^ to inhibit lipid peroxidation and malondialdehyde (MDA) formation; and 3) recovering mitochondrial function as a reactive oxygen species (ROS) scavenger. Created with BioRender.com.

We confirmed that ferroptosis partially participates in IVDD according to the GEO database analysis. IVDD or herniation has a considerable effect on society, leading to sub‐health status (even disability); we used PDA NPs for IVDD management in vivo and in vitro, which provides a novel and biocompatible method to suppress the degeneration and even regenerate intervertebral discs by targeting ferroptosis. The underlying mechanism depends on three different pathways, namely 1) scavenging ROS and chelating Fe^2+^ ions, 2) preventing downstream activation of the peroxidation reaction (which accelerated the production of OH and accumulation of MDA and LPO), and interestingly, 3) suppressing the ubiquitin‐mediated degradation of GPX4 and increasing its expression functions as a lipid repair enzyme^[^ ^39^ ^]^ (**Figure** **7**). Exerting these three effects, PDA NPs successfully inhibited ferroptosis and degeneration in a puncture‐induced rat IVDD model in vivo (Figure 6) and the TBHP‐induced degenerated NP cell model in vitro (Figure 3).

Previous studies have shown that ferroptosis is involved in the mechanisms of IVDD in humans,^[^ ^22^, ^40^, ^41^ ^]^ and the KEGG analysis of GSE56081 in our study also partially demonstrated that it is an etiology of degeneration, consequentially indicating the necessity of treating IVDD by targeting ferroptosis. The contribution of ferroptosis during IVDD was also validated in another dataset, GSE27494, which further demonstrated the importance of ferroptosis in degeneration mechanisms (Figure S1, Supporting Information). Moreover, we demonstrated that PDA exerted therapeutic effects by targeting ferroptosis during IVDD, providing evidence for the involvement of ferroptosis during IVDD. Hub genes imported into GeneMANIA (http://genemania.org/) for additional function predictions revealed the close relation of IVDD with metal ion metabolism (Figure S1, Supporting Information). Furthermore, the Area Resources for Community and Human Services (ARCHS) public database (https://maayanlab.cloud/archs4/index.html) showed that abnormal expression levels of GPX4 in patients were closely associated with many human phenotypes, including “abnormal activity of mitochondria respiratory chain” and “decreased activity of mitochondria respiratory,” implying the importance of balanced GPX4 expression for mitochondrial function (Table S3, Supporting Information). Combined with our GSE56081 analysis, the association of GPX4 downregulation and mitochondrial structural disorder and dysfunction was in accordance with our in vitro and in vivo study results (Figures 4 and 6), and presents a potential therapeutic target to further explore the potential involvement of GPX4 in the crosstalk between ferroptosis and IVDD.

Ferroptosis was first reported by Dr. Brent R Stockwell in 2012, stating that it is a unique modality of cell death, driven by iron‐dependent phospholipid peroxidation.^[^ ^42^, ^43^ ^]^ Usually initiated by oxidative stress,^[^ ^44^ ^]^ the critical process of ferroptosis is Fe^2+^ accumulation, ROS production, and finally, lipid peroxides and MDA up‐regulation.^[^ ^45^ ^]^ However, several other intrinsic or exogenous pathways also induce ferroptosis,^[^ ^46^, ^47^ ^]^ including the system xc^−^ cystine–glutamate antiporter to absorb cystine, an ingredient of GSH synthesis.^[^ ^48^ ^]^ Subunits of system xc^−^, Solute Carrier Family 7, Member 11 (SLC7A11) and Solute Carrier Family 3, Member 2 (SLC3A2), combined with thioredoxin reductase 1 (TXNRD1) and GPX4, are the key genes functioning during this process^[^ ^49^, ^50^, ^51^, ^52^ ^]^ and blockade of these proteins could effectively break down the transformation of phospholipid hydroperoxides (PL‐OOH) into the corresponding alcohols (P‐LOH),^[^ ^53^ ^]^ inducing ferroptosis. In our study, we found that PDA NPs administration assisted hemostasis maintenance via GPX4 upregulation in a post‐translational modification way (Figure 4). Moreover, in another pathway of ferroptosis‐related metabolism of Fe^2+^,^[^ ^54^ ^]^ PDA NPs significantly reduced ferritin and FHC expression and antagonized the deterioration function of TBHP to rescue the reduction of these iron storage proteins in NP cells in vitro.^[^ ^55^ ^]^ Consistent with previous studies,^[^ ^56^ ^]^ maintaining the ferritin and FHC and reducing the increase in Xct and TFR^[^ ^57^ ^]^ (stimulated by TBHP) can prevent ferroptosis in cells.

GPX4 is a key ferroptosis regulator of ferroptosis. Many bioactive compounds isolated from herbs^[^ ^58^ ^]^ or small molecules^[^ ^59^ ^]^ have been identified as ubiquitination inducers targeting GPX4 to activate ferroptosis in cancer cells. Thus, the post‐translational modification of GPX4 has received increasing scientific attention.^[^ ^60^ ^]^ Here, we first proved that the ubiquitin‐mediated degradation of GPX4 exists in NP cells (Figure 4). Different from the previous studies on PDA NPs,^[^ ^31^, ^61^ ^]^ we delved deeper into the protein–nanoparticles connection and interaction by ubiquitination assay: The PDA NPs were transferred into the NP cell cytoplasm via endocytosis (co‐localized with endosome marker rab5, Figure 5) and assembled near the mitochondria (co‐localized with Mito‐tracker, Figure 5), where it then interacted with GPX4 to inhibit ubiquitin mediated degradation, ultimately functioning as a ferroptosis suppressor (Figures 4 and 5). However, the direct interaction sites and mechanisms require further elucidation in future studies.

We used the canonical puncture‐induced degeneration rat model to support the conclusion that PDA NPs suppress ferroptosis effectively in vivo. In the short period, the needle puncture penetrates through the annulus fibrosus; it destroys the special micro‐environment of the intervertebral discs, resulting in a poor blood supply and low oxygen concentration.^[^ ^62^, ^63^, ^64^, ^65^ ^]^ Over time, the fibrotic repair process embargoes with angiogenesis distributing into the disc.^[^ ^66^ ^]^ Apart from the increased inflammatory cytokines following penetration,^[^ ^67^, ^68^ ^]^ excessive blood and oxygen accumulate in the discs, causing a ROS burst,^[^ ^69^ ^]^ which in turn stimulates further peroxidation of lipids,^[^ ^24^ ^]^ proteins, and even nucleic acids,^[^ ^70^ ^]^ leading to detrimental signaling pathway activation.^[^ ^71^ ^]^ During this process, the hemoglobin from the newly‐formed blood vessel increases in the disc, inducing an overload of Fe ions and ferroptosis of NP cells after being metabolized and degraded.^[^ ^23^ ^]^ Previously, we demonstrated that puncture increases ubiquitin levels in NP cells.^[^ ^72^ ^]^ In addition, the anti‐ferroptosis protein, GPX4, was decreased after a puncture in previous studies^[^ ^41^, ^73^ ^]^ which is consistent with our results (Figure 6). To conclude, PDA NPs administration can effectively restore GPX4 expression and inhibit ubiquitin‐mediated degradation in the discs in vivo, further confirming the protective mechanisms of PDA NPs (Figures 4, 5, 6).

Some deficiencies need to be optimized in future studies. Safer and less dosage is expected to apply for more effective treatment. Moreover, repeated injection in the disc may induce degeneration; more sustainable nanoparticles will be explored in vivo therapy.

## 4 Conclusion

A novel strategy targeting ferroptosis for the treatment of intervertebral disc degeneration was proposed in this work by the PDA NPs administration. The in vitro study showed that PDA NPs assisted cell homeostasis maintenance and antagonized oxidative stress‐induced ferroptosis of NP cells by collaborative ROS depletion, Fe^2+^ accumulation reduction, and more importantly, the ubiquitination suppression of GPX4. Our study demonstrated that PDA NPs interact with GPX4 for post‐translational modification, and exploring this mechanism provides new insights regarding protein–material interactions, which will be relevant for further studies. Moreover, it offers a new target of ferroptosis, which mitigates intervertebral disc degeneration, which holds the potential to contribute to clinical IVDD therapeutics in the future.

## 5 Experimental Section

### Bioinformatics Analysis

The mRNA expression profile dataset GSE56081 was downloaded from the GEO database (https://www.ncbi.nlm.nih.gov/geo/) in the data format MINiML and subjected to identity document (ID) transformation, expression normalization, and principal component analysis (PCA). Genes with *P* value < 0.05 were considered as differentially expressed genes (DEGs) using “limma” package in R. KEGG pathway enrichment analysis was conducted based on DEGs by the “ClusterProfiler” package and the cutoff criteria of “*P* value (from the Benjamini–Hochberg method), 0.05” was considered as statistically significant. The PPI network of DEGs was further analyzed using the STRING database and results were further imported into Cytoscape software (version 3.9.1) with the top 5% scores as hub genes analyzed by Cytohubba plugin. The correlation of hub genes expression in each sample was then analyzed and exhibited by heatmap in the formation of circline. The results above including PCA, boxplot, and heatmap were further exhibited using the R package “ggord,” “ggplot2,” and “circlize”.

### Intervertebral Discs Specimens

Intervertebral discs (≥5 mm) obtained from eight patients were divided into two groups: The control group consisted of patients diagnosed with congenital spondylolisthesis and congenital scoliosis (two males and two females with an average age of 11.75 ± 2.062 years; age ranges from 9 to 14 years). The IVDD group consisted of patients diagnosed with lumbar spinal stenosis (LSS), degenerative lumbar spondylolisthesis (DLS), and lumbar disc herniation (LDH) (two males and two females with an average age of 66.5 ± 10.02 years; age ranges from 57 to 79 years) (Table S1, Supporting Information). The samples were subjected to immunohistochemical analysis. The degree of intervertebral disc degeneration was evaluated using the Pfirrmann grading system.

### Preparation of PDA NPs

Polydopamine nanoparticles (PDA NPs) were prepared according to the literature.^[^ ^30^ ^]^ Typically, ethanol (80 mL) was added into ammonium hydroxide solution (200 mL, 0.55% wt) and stirred for 1 h at room temperature. Then, dopamine hydrochloride solution (0.05 g mL^−1^, 20 mL) was added into the above mixture, and the reaction was allowed to proceed under stirring for 24 h. Last, PDA NPs were obtained via centrifugation, washed with water, and dispersed in deionized water for further use.

To label the PDA NPs, the NPs (1 mg mL^−1^) were activated by N‐hydroxysuccinimide (NHS)/Dopamine hydrochloride, 1‐ethyl‐3‐(3‐(dimethylamino) propyl) carbodiimide (EDC) (100 mm; Sigma‐Aldrich, USA). Then, 5 mol. equiv. of NH_2_‐PEG‐COOH (*M*_w_ = 2000 Da; Confluore Biotech, Inc. Xi An, China) was added to the PDA NPs solution and the mixture was stirred overnight. After centrifugation and washing repeatedly with deionized water, the PDA NPs were incubated with fluorescein isothiocyanate (FITC; 1 mg mL^−1^; Aladdin Biochemical Technology Co., Ltd, China) at room temperature overnight. The FITC labeled PDA NPs (denoted as FITC‐PDA) were collected by centrifugation at 12 000 rpm for 10 min and repeatedly washed with deionized water.

### Characterization of the PDA NPs

The particle size and morphology of the PDA NPs were characterized by transmission electron microscopy (TEM, JEM‐2100F, Japan) and scanning electron microscopy (SEM, SU8020, Hitachi, Japan). The hydrodynamic size was measured using dynamic light scattering (DLS, Zeta Plus, Brookhaven, USA). Ultraviolet‐visible (UV–vis) absorption spectra were recorded on a UV–Vis–NIR spectrophotometer (UV‐3600, Shimadzu, Japan). Electron spin resonance (ESR) spectroscopy was carried out using an ESR spectrometer (Bruker EMX, USA).

### Determination of the ROS Scavenge Capacity of PDA NPs

The antioxidant capacity of PDA NPs was initially investigated using the 2,2'‐azino‐bis (3‐ethylbenzthiazoline‐6‐sulfonic acid (ABTS) method according to a total antioxidant capacity assay kit (Beyotime Technology). Briefly, different concentrations of PDA NPs (25, 50, 100, 200, and 500 µg mL^−1^) were added into the ABTS working solution and the mixture was incubated in the dark at ambient temperature for 10 min. After filtration by a micropore filter membrane (0.22 µm), the absorbance intensity at 415 nm was recorded by UV–vis spectroscopy (*n* = 3 for each group).

The hydroxyl radicals (•OH) and superoxide anions (O_2_^•−^) depletion capacity of PDA NPs was investigated by electron spin resonance (ESR) assays. In brief, •OH was generated by incubating FeSO_4_ (0.4 mm) and H_2_O_2_ (10 mm) for 20 min at ambient temperature. Then, PDA NPs (0, 50, and 200 µg mL^−1^) were added to the •OH‐containing solution. The •OH signals were captured by an ESR spectrometer using 5,5‐dimethyl‐1‐pyrroline N‐oxide (DMPO) as the spin probe. Similarly, O_2_^•−^ was generated by a xanthine/xanthine oxidase system containing 0.05 mm diethylenetriaminepentaacetic acid (DTPA), 1 mm xanthine, and 0.2 U mL^−1^ xanthine oxidase. Then, the ability of PDA NPs (0, 50, and 200 µg mL^−1^) for the quenching of O_2_^•−^ was investigated by an ESR spectrometer using DMPO as a O_2_^•−^ trapping agent.

The H_2_O_2_ depletion capacity of PDA NPs was then evaluated using a colorimetric titanium sulfate assay kit (Solarbio Life Sciences). Briefly, H_2_O_2_ solutions (1 mm) were incubated with PDA NPs (0, 50, 100, and 500 µg mL^−1^) for 1 h at room temperature. After filtration by a micropore filter membrane (0.22 µm), the concentrations of H_2_O_2_ in the filtrate were measured by monitoring the absorption of peroxide‐titanium at 410 nm using the UV–vis spectroscopy (*n* = 3 for each group). Then, the degradation of PDA NPs in H_2_O_2_ solution (5 and 10 mm) was monitored by UV–vis spectroscopy. Briefly, 5 and 10 mm of H_2_O_2_ solution was added into the PDA NPs solution (100 µg mL^−1^) and the mixture was kept in the dark at room temperature for 24 h. Afterward, the UV–vis absorption spectra of the mixture was recorded.

### Determination of the Iron‐Chelating Capacity of PDA NPs

The Fe^2+^‐chelating capacity of PDA NPs was investigated by the ferrous ion content assay kit (Solarbio Life Sciences). In brief, FeSO_4_ solution (100 µm) was incubated with PDA NPs (25, 50, 100, 200, and 500 µg mL^−1^) for 1 h at room temperature, respectively. The mixture was filtrated by a micropore filter membrane (0.22 µm), and the concentrations of Fe^2+^ in the filtrate were measured by monitoring the absorbance of blue tripyridyltriazine‐Fe^2+^ complex at 600 nm via UV–vis spectroscopy (*n* = 3 for each group). The Fe^2+^‐chelating capacity of PDA NPs was calculated according to the standard curve of FeSO_4_ solution. Then, to determine the Fe^3+^‐chelating capacity of PDA NPs, the PDA NPs (25, 50, 100, 200, and 500 µg mL^−1^) were mixed with Fe^3+^ standard solution (125 µm) for 4 h at room temperature. The mixture was filtrated by a micropore filter membrane (0.22 µm), and the concentrations of Fe^3+^ in the filtrate were measured and calculated by monitoring the absorbance of 2,2'‐ bipyridine‐Fe^3+^ complex at 520 nm via UV–vis spectroscopy (*n* = 3 for each group).

### Culture of NP Cell and 293T Cell

The NP cell line of rats was gifted by Dr. Chen Di kindly at the Department of Orthopedic Surgery, Rush University Medical Center (Chicago, IL, USA). 293T cells are immortalized cell lines purchased from Shanghai Fuheng Biological Company (cat. no. FH0244). These two cells were cultured in Dulbecco's modified Eagle's medium (DMEM) supplemented with 10% FBS and 1% penicillin–streptomycin (Gibco, Thermo Fisher Scientific, Waltham, MA, USA) at 37 °C with 5% CO_2_.

### Cell Viability Analysis and Cytotoxicity Assay

Cell viability and cytotoxicity following different ranges of concentrations of PDA NPs treatment (0, 0.25, 0.5, 1, 10, and 20 µg mL^−1^) were evaluated according to the cell counting kit‐8 (CCK‐8; Dojindo Laboratories Co., Ltd, Kumamoto, Japan). NP cell line was seeded onto a 96‐well plate at a density of 2 × 10^3^ cells per well and then was treated with increasing concentrations of PDA NPs for 24, 48, and 72 h the next day (for the cytotoxicity assay, the seeded number was 8 × 10^3^ cells per well for 24 h), cultured in DMEM supplemented with 10% FBS and 1% penicillin/streptomycin (complete DMEM). Cell media was changed every 2 days. At the experimental ending periods, cells were incubated with complete media containing 10 µL of CCK‐8 for 2 h at 37 °C. The same media containing CCK‐8 without cells or with untreated cells was applied as blank and mock controls, respectively (*n* = 6 for each group). The absorbances (measured as optical density; OD) at 450 nm were measured using an Infinite M200 Pro multimode microplate reader (Tecan Life Sciences, Männedorf, Switzerland).

### High‐Density Culture

1.5 × 10^5^ NP cells were resuspended in 10 µL medium and seeded in a 24‐well plate. Cells were cultured at 37 °C for 1 h, then 0.5 mL DMEM/F12 medium containing 10 ng mL^−1^ insulin–transferrin–selenium (ITS) and 2% FBS was added. After 24 h at 37 °C, the cells were stimulated with TBHP (100 µm, 458139; Sigma–Aldrich, USA) and/or PDA NPs. The medium was changed every 2 days. After 9 days at 37 °C, the micro‐mass was stained with alcian blue for 24 h at room temperature (RT; *n* = 3 for each group).

### Dead/Live Cells Assay

NP cells were seeded into confocal dishes and incubated with PDA NPs or TBHP (100 µm) in the presence/absence of PDA NPs, followed by incubation with 1 mL of Calcein/PI working buffer for 30 min at 37 °C according to the Calcein/PI stain Assay Kit (no. C2015, Beyotime, China). The intensity of dead/live cells (PI/Calcein) was examined by the Leica DM4000 B epifluorescence microscope (*n* = 3 for each group) and the rate of IOD/DAPI was carried out using Image‐Pro Plus 6.0 software (Media Cybernetics, Inc.).

### Intracellular GSH, MDA, and Iron Assay

NP cells were stimulated with TBHP (100 µm) for 12 h pretreated with or without PDA NPs (1 µg mL^−1^) for 24 h at 37°C with 5% CO_2_. The amount of GSH was measured via the GSH Assay Kit standardized via the weight of cells; the MDA amount was evaluated using the MDA Assay Kit standardized via the weight of cells; the iron amount (Fe^2+^, Fe^3+^) was monitored using Iron Assay Kit. All reagents used here were purchased from Dojindo Laboratories Co., Ltd, Kumamoto, Japan (*n* = 3 for each group).

### Intracellular Fe^2+^, ROS, and Lipid Peroxidation (LPO) Detection

NP cells were seeded into confocal dishes and stimulated with TBHP (100 µm) with/without PDA NPs, then incubated with 2 µL of FerroOrange (1 µm), DCFH‐DA (10 µm), and Liperfluo probe (10 µm) for 30 min at 37 °C. Last, the change of Fe^2+^, ROS, and LPO was examined by the Leica DM4000 B epifluorescence microscope (Leica Microsystems), after which the intensity and IOD/DAPI were carried out using Image‐Pro Plus 6.0 software (Media Cybernetics, Inc.). Another examination method was to analyze the intensity using flow cytometry. All reagents used here were purchased from Dojindo Laboratories (*n* = 3 for each group).

### RNA Extraction and Real‐Time Quantitative PCR (qPCR) Analyses

NP cells were stimulated with different concentrations of PDA NPs (0, 0.25, 1 µg mL^−1^, dissolved in PBS). As for the TBHP treatment, NP cells were pretreated with or without PDA NPs for 24 h; then, cells were exposed to TBHP (100 µm) treatment for 12 h. Then, total RNAs were extracted using TRIzol reagent (Thermo Fisher Scientific, Waltham, MA, USA) according to manufacturer's protocol. This was followed by the reversed transcription to first‐strand complementary DNAs (cDNAs) from RNAs via the cDNA Synthesis Kit (Takara Bio, Otsu, Japan). Then, real‐time qPCR was conducted on an Applied Biosystems QuantStudio 6 Flex Real‐Time PCR System (Thermo Fisher Scientific) using the TB Green Premix Ex Taq Kit (Takara Bio) with the following protocols: denaturation at 95 °C, 30 s; 40 cycles of 95 °C, 3 s and 60 °C, 34 s; and then, 95 °C, 15 s, 60 °C, 60 s, and last of all, 95 °C, 15 s. NCBI BLAST was used to design specific primer pairs and then the sequences were provided in Table S2, Supporting Information. *β*‐actin was used as the internal control. Target gene expression levels were determined using the 2^−ΔΔCT^ method (*n* = 3 for each group).

### Western Blot Analysis

NP cell line was treated with different concentrations of PDA NPs (0, 0.25, 1 µg mL^−1^, dissolved in PBS) or stimulated with TBHP (100 µm) for 12 h pretreated with PDA NPs (1 µg mL^−1^) for 24 h. Then, total proteins were isolated from cells using RIPA lysis buffer supplemented with phosphatase and protease inhibitors (M5293, M7528; Abmole, China). Following quantification by BCA assay (Thermo Fisher Scientific, Inc.), equal quantities of extracted proteins (20–30 µg) were added on 10% or 12.5% SDS‐PAGE gel for electrophoresis and then separated proteins were electroblotted onto 0.22 µm PVDF membranes (Merck‐Millipore). Membranes were blocked with 5% BSA‐PBS at RT for 1 h and then incubated with primary antibodies (diluted 1:1000 in 5% BSA‐PBS) overnight at 4 °C (at least 16 h) after washing thrice with TBST. Primary antibodies against ferritin (cat. no. ab75973; rabbit mAb), GPX4 (cat. no. ab125066; rabbit mAb), TFR (cat. no. ab214039; rabbit mAb), FHC (cat. no. ab183781; rabbit mAb), Xct (cat. no. ab175186; rabbit mAb), and *β*‐actin (cat. no. D6A8; rabbit mAb) were purchased from Abcam (Cambridge, UK) and Cell Signaling Technology (Danvers, MA, USA). Membranes were then washed in Tris‐buffered saline‐Tween20 (TBST) and incubated with secondary antibody anti‐rabbit IgG (H+L) (DyLight 800 4× PEG Conjugate; Cell Signaling Technology, 1:5000 dilution) for 1 h at RT in the dark. Membranes were again washed in TBST and then were detected on an LI‐COR Odyssey Fluorescence Imaging System (LI‐COR Biosciences, Lincoln, NE, USA). Semi‐quantitative analysis of protein band intensity was measured using ImageJ V1.8.0 software (National Institutes of Health) and normalized to the internal control *β*‐actin (*n* = 3 for each group).

### OXPHOS Analysis

NP cell line was treated with PDA NPs alone (1 µg mL^−1^, dissolved in PBS) for 24 h or stimulated with TBHP (100 µm) for 12 h pretreated with or without PDA NPs (1 µg mL^−1^) for 24 h at 37 °C with 5% CO_2_. Then, total proteins were isolated from cells using RIPA lysis buffer supplemented with phosphatase and protease inhibitors (M5293, M7528; Abmole, China). Following quantification by BCA assay (Thermo Fisher Scientific, Inc.), being added with 5× protein loading buffer and being boiled at 60 °C for 10 min, equal quantities of extracted proteins (20–30 µg) were subjected to WB analysis. Primary antibodies against OXPHOS (cat. no. ab110413; mouse mAb) were bought from Abcam (Cambridge, UK) (*n* = 3 for each group).

### Ubiquitylation Modification Assay

293T cells and NP cells were transfected with Flag‐PLVC, Myc‐Ubiquitin, and Flag‐GPX4 plasmids (synthesized, purchased from Shanghai Ai Bosi Biological Technology Co., Ltd.) using lipofectamine 3000 (cat. no. L3000015; Thermo Fisher Scientific, Inc., Waltham, MA, USA) according to manufacturer's protocol. After 40 h, cells were treated with MG132 (10 µm; cat. no. S2619; Selleck Chemicals, China) for 8 h at 37 °C and then washed three times with PBS. Proteins were extracted using 1.2 mL NETT and then centrifuged to discard precipitation. 200 µL lysate was used as input and the remaining 1000 µL lysates were incubated with 30 µL flag‐tagged magnetic beads at 4 °C overnight. The samples were then boiled at 99 °C for 10 min and finally subjected to immunoblot analysis (*n* = 3 for each group).

For in vivo ubiquitylation assay, the lysates were quantified by BCA assay and then diluted to the same protein concentration. Then, 100 µL lysates were used as input and 300 µL lysates were incubated with 30 µL A/G protein magnetic beads (which were pre‐incubated with mouse anti‐GPX4 [cat. no. 67763‐1‐Ig; Protein‐tech] at RT for 15 min) at 4 °C overnight. The samples were then boiled at 99 °C for 10 min and finally subjected to immunoblot analysis (*n* = 3 for each group).

### Transmission Electron Microscopy

Morphological changes in the cell were monitored via electron microscopy (*n* = 6 for each group). After being treated with 100 µm TBHP for 12 h and pretreated with or without PDA NPs for another 12 h, NP cells were dissociated and fixed in 2.5% ice‐cold glutaraldehyde overnight, postfixed in osmium tetroxide, and dehydrated with a series of alcohol concentrations. After that, the cells were rinsed with propylene oxide and impregnated with epoxy resin. Uranyl acetate and lead citrate were used by microscopy as negative control. Electron micrographs were obtained by the Hitachi transmission electron microscope (TEM) system according to the manufacturer's protocol (HC/HR select = HC‐1, Accelerating voltage = 80 000, Emission = 10.2, Vacuum = 5.6E‐05 Pa).

### Seahorse Assay

NP cells were seeded into XF‐96 cell culture plates at a density of 5 × 10^3^ per well (Seahorse Bioscience, USA). Then, the cells were treated with PDA NPs alone for 24 h or stimulated with TBHP (100 µm) for 12 h pretreated with or without PDA NPs for 24 h. Oxygen consumption rate (OCR) and extracellular acidification rate (ECAR) were measured according to standard protocols, and the results were analyzed via Seahorse XF‐96 Flux Analyzer (Seahorse Bioscience). For the OCR assay, cells were stimulated with the following 1.5 µm oligomycin, 2.5 µm FCCP/rotenone, and 0.5 µm antimycin A. In the EACR assay, cells were stimulated with 10 mm glucose, 2 µm oligomycin, and 50 mm 2‐DG (*n* = 3 for each group).

### Animals and Surgical Procedures

All animal experiments were approved by the Institutional Animal Care and Ethics Committee of Ninth People's Hospital, Shanghai Jiaotong University School of Medicine (Shanghai, China), and performed per the principles and procedures of the National Institutes of Health (NIH) Guide for the Care and Use of Laboratory Animals and the Guidelines for Animal Treatment of Shanghai Jiao Tong University.

Six male 8‐week‐old Sprague‐Dawley rats (Shanghai Lab, Animal Research Center Co. Ltd, Shanghai, China) were housed under pathogen‐free conditions at 26–28 °C and 50–65% humidity with 12 h day/night cycle. Animals were fed standard rodent chow and had access to fresh water ad libitum. Before surgical procedures, rats were anesthetized by intraperitoneal injections of pentobarbital sodium (5 mg per 100 g of body weight). Tails were sterilized with iodinated polyvinylpyrrolidone and then a longitudinal skin incision was made on the right side of the tail to show the intervertebral disc at coccyx vertebrae 6–10. The intervertebral disc at Co6/7 was Sham control and the intervertebral discs at Co7/8, Co8/9, and Co9/10 were punctured with a 20‐gauge sterile needle. The needle was oriented perpendicular to the skin to make penetration at the center of the disc, rotated 360°, and kept in the disc for 15 s. Then, the 10 µL of PDA NPs at concentrations of 0.25 and 1 µg mL^−1^ were injected, respectively into intervertebral discs at Co8/9 and Co9/10 via a 33‐gauge needle connected to a 100‐µL micro‐syringe (MICROLITER Series. 700; Hamilton Bonaduz, Switzerland). After the syringe was taken out from the disc, the incision was then sutured. The rats were treated with PDA NPs once a week via a cutaneous incision in the following 2 weeks and were allowed another 2 weeks for recovery. At the end period, all rats were sacrificed with tails extracted and fixed in 4% PFA.

For in vivo PDA NPs degradation assay, 12 male 8‐week‐old Sprague‐Dawley rats (divided into four groups, *n* = 3, 0 h, 48 h, 7 d, and 14 d) were anesthetized by intraperitoneal injections of pentobarbital sodium (5 mg per 100 g of body weight). Tails were sterilized with iodinated polyvinylpyrrolidone and then a longitudinal skin incision was made on the right side of the tail to show the intervertebral disc at coccyx vertebrae 6–9. The intervertebral discs at Co6‐9 were administered 10 µL PDA‐NPs/Cu^2+^ at a concentration of 1 mg mL^−1^; then, at the end of the period, the rats were sacrificed and the discs were extracted for inductively coupled plasma mass spectrometry (ICP‐MS, NexION 2000, Unit: PPm) assay of the concentration of Cu^2+^. The 0 h was used as the control group and the percentage compared with the control group was calculated.

For in vivo ubiquitination assay, 12 male 8‐week‐old Sprague‐Dawley rats (divided into four groups and *n* = 3: control, puncture, puncture + 0.25 µg mL^−1^ PDA NPs, and puncture + 1 µg mL^−1^ PDA NPs) were anesthetized by intraperitoneal injections of pentobarbital sodium (5 mg per 100 g of body weight). Tails were sterilized with iodinated polyvinylpyrrolidone and then a longitudinal skin incision was made on the right side of the tail to show the intervertebral discs at coccyx vertebrae 6–9. The intervertebral discs at Co6‐9 were intact in the control group and punctured in the other three groups using 20G needles. For 0.25 and 1 µg mL^−1^ PDA NPs groups, the discs were administered with PDA‐NPs, respectively. Then at the end of the period, the rats were sacrificed and the discs were extracted, grinding with liquid nitrogen, and lysed with RIPA buffer for subsequent ubiquitylation assay.

### Histology and Immunofluorescence Staining

Fixed intervertebral discs (*n* = 6 for each group) were embedded into paraffin, then subjected to histological sectioning (8 µm thickness). For histological assessment, the paraffin sections were de‐paraffinized in graded xylene, rehydrated in graded alcohol solutions, and washed and stained with Safranin O‐Fast Green and hematoxylin and eosin (H&E) staining (Servicebio, Wuhan, China) per the manufacturer's protocols; the histological score was calculated based on the modified histologic grading system.^[^ ^33^ ^]^ For immunofluorescence staining, prepared paraffin sections were incubated in antigen retrieval buffer (Roche Diagnostics) at 37 °C for 30 min. After cooling to RT, the sections were washed with PBS three times for 5 min each, Then, an auto‐fluorescence quencher was added to the sections for 5 min and blocked for 30 min with a blocking buffer at RT. Sections were then incubated with primary antibodies at 4 °C overnight at 1:100 dilution, including anti‐GPX4 (cat. no. 67763‐1‐Ig; Protein‐tech), anti‐ubiquitin (cat. no. 10201‐2‐AP; Protein‐tech). The next day, sections were washed and then incubated with Alexa Fluor 488 and 555 Conjugate secondary antibody (anti‐rabbit, anti‐mouse, 1:500; Cell Signaling Technology) for 50 min at room temperature in the dark, washed with PBS, and incubated with DAPI solution (Sigma–Aldrich, St Louis, MO, USA) for 10 min in the dark. Finally, sections were subjected to final washes, air‐drying, and then processed with anti‐fluorescence quenching tablets. Fluorescence images were captured using a Leica DM4000 B epifluorescence microscope (Leica Microsystems) and IOD/DAPI measurements were carried out using Image‐Pro Plus 6.0 software (Media Cybernetics, Inc.).

For the Mito‐tracker assessment of NP cell‐line, cells were seeded onto a confocal dish. At 10% confluence, the cells were stained with live cell stain Hoechst 333 and Mito‐tracker (Beyotime Technology) at RT for 15 min and then captured using a Leica DM4000 B epifluorescence microscope (*n* = 3 for each group).

For endocytosis and GPX4 assessment of the NP cell line, cells were seeded onto a confocal dish. At 10% confluence, the cells were processed as sections as aforementioned, of which the primary antibodies were anti‐GPX4 (cat. no. 67763‐1‐Ig; Protein‐tech), anti‐Rab5 (cat. no. ab218624; rabbit mAb; Abcam) (*n* = 3 for each group).

### Immunohistochemistry

Fixed intervertebral disc tissue samples and human samples were embedded in paraffin and cut into slices (8 µm), then processed in an immunohistochemistry kit (cat. no. G1215‐200T; Wuhan Servicebio Technology Co., Ltd.) per the manufacturer's instructions. The primary antibody included Anti‐GPX4 (cat. no. 67763‐1‐Ig; Protein‐tech). Digital images were captured using a Leica DM4000 B microscope, and the ratio of the positively‐stained cell was calculated using Image Pro Plus 6.0 software.

### Radiographic and Magnetic Resonance Imaging (MRI) Analysis

Digital X‐ray imaging of the punctured intervertebral discs was captured in the anteroposterior axis with a 21 lp mm^−1^ detector, up to 5× geometric magnification (Faxitron VersaVision; Faxitron Bioptics LLC, Tucson, AZ. USA). MRI imaging of the same punctured intervertebral discs was carried out on a Siemens Magnetom Prisma E11 (Siemens Healthineers, Erlangen, Germany) with the following parameters: TR 3000 ms, TE 80 ms, 1.1 mm thickness, 0.22 mm interval, FOV 160 × 65 mm, and voxel size 0.25 × 0.25 × 1.1 mm.

### Statistical Analysis

Independent experiments or repeated measurements were conducted for all data (*n* = 3, 4, or 6, respectively). Data were displayed as mean ± standard deviation (SD). Significance differences between groups were calculated by one‐way analysis of variance (ANOVA) with Tukey's post hoc test. Significant differences in ordinal data were assessed by Kruskal–Wallis with Dunn's post hoc test. Analysis was conducted using SPSS 19.0 software (IBM Corporation, Armonk, NY, USA). The difference was considered significant if the *p‐*value was less than 0.05 (* indicates *P* < 0.05, ** indicates *P* < 0.01, *** indicates *P* < 0.001, and **** indicates *P* < 0.0001).

### Ethic Statement

Human tissues mentioned above were extracted as waste material from the surgery performed at the Shanghai Ninth People's Hospital, and the written informed consent for the use of the tissues was obtained from the patients in Table S1, Supporting Information. Human Ethics approval was received from the Institutional Human Ethics Review Board of the Shanghai Ninth People's Hospital, Shanghai Jiao Tong University School of Medicine (Approval#SH9H‐2021‐T94‐2). Animal ethics approval was received from the Institutional Animal Ethics Review Board of Shanghai Ninth People's Hospital, Shanghai Jiao Tong University School of Medicine (Approval no. SH9H‐2021‐A607‐SB).

## Conflict of Interest

The authors declare no conflict of interest.

## Supporting information

Supporting Information

## Data Availability

The data that support the findings of this study are available from the corresponding author upon reasonable request.
