# Supplementary material for: Polydopamine Nanoparticles Targeting Ferroptosis Mitigate Intervertebral Disc Degeneration Via Reactive Oxygen Species Depletion, Iron Ions Chelation, and GPX4 Ubiquitination Suppression
Source: Adv Sci (Weinh). 2023 Mar 23;10(13):2207216. doi: 10.1002/advs.202207216 (PMC10161035; doi:10.1002/advs.202207216)
Supplement: Supplementary file 1 — Supporting Information [file ADVS-10-2207216-s001.pdf]

Supporting Information

Polydopamine Nanoparticles Targeting Ferroptosis Mitigate Intervertebral Disc Degeneration via Reactive Oxygen Species Depletion, Iron Ions Chelation and GPX4 Ubiquitination Suppression

Xiao Yang[1a], Yan Chen[1a], Jiadong Guo[1a], Jiaxin Li[3], Pu Zhang[1], Huan Yang[1, 2], Kewei Rong[1], Tangjun Zhou[1], Jingke Fu[1*], Jie Zhao[1*]*

[1]Shanghai Key Laboratory of Orthopedic Implants, Department of Orthopedics, Ninth People's Hospital, Shanghai Jiao tong University School of Medicine, 639 Zhizaoju Road, Shanghai, 200011, P. R. China

[2]The Second Clinical Medical College of Yunnan University of Traditional Chinese Medicine, 1076 Yuhua Road, Kunming, 650500, China.

[3]Department of Orthopedics, The Second Affiliated Hospital of Harbin Medical University, 246 Xuefu Road, Harbin, 150001, China.

[a]These authors contributed equally to this work.

Email addresses:

Xiao Yang: walkingweapon@outlook.com;
Yan Chen: chenyortho@163.com
Jiadong Guo: 1226837284@qq.com;
Jiaxin Li: YXlijiaxin163@163.com:
Pu Zhang: 2205237465@qq.com;
Huan Yang: 2547818826@qq.com;
Kewei Rong: rick_long@sjtu.edu.cn

***Correspondence:**

Tangjun Zhou: zhoutangjun@outlook.com

Jingke Fu: fujingke@sjtu.edu.cn
Jie Zhao: profzhaojie@126.com

Keywords: ferroptosis, intervertebral disc degeneration, nucleus pulposus, polydopamine nanoparticles, GPX4 ubiquitination

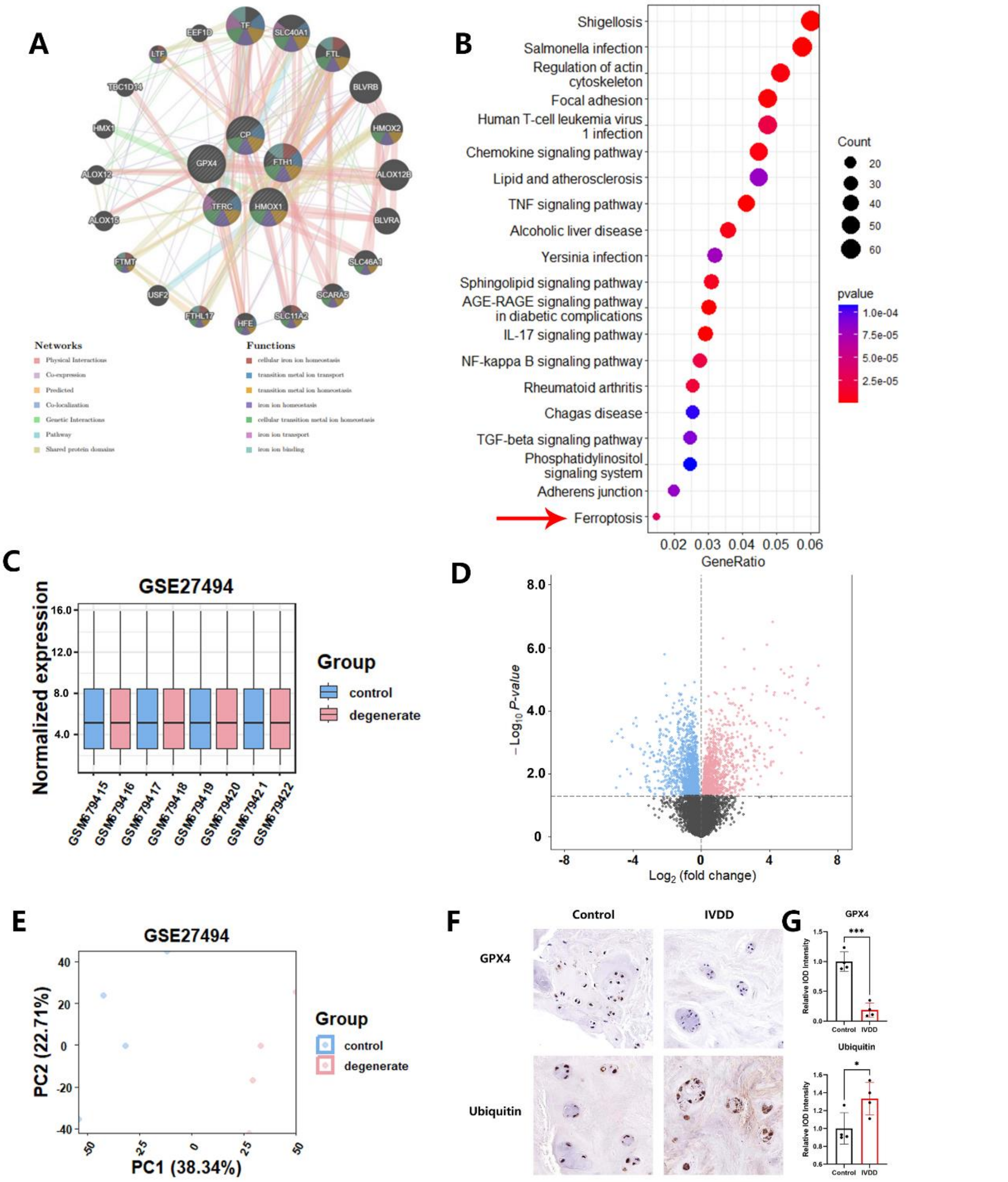

Supplementary Figure 1. (A) The interaction among CP, FTH1, GPX4, HMOX1, and TFRC was analyzed by GeneMANIA. (B) KEGG pathway enrichment analysis of GSE27494. (C) Normalized expression matrix of GSE27494 demonstrated by a boxplot. (D) The volcano plot of GSE27494. (E) Principal component analysis (PCA) diagram of GSE27494. (F and G) Expression and quantification of GPX4 and Ubiquitin in the human IVDD samples compared with control validated by IHC. All data are presented as mean ± standard deviation (SD) from four replicates. *P<0.05, **P<0.01, ***P<0.001, and ****P<0.0001.

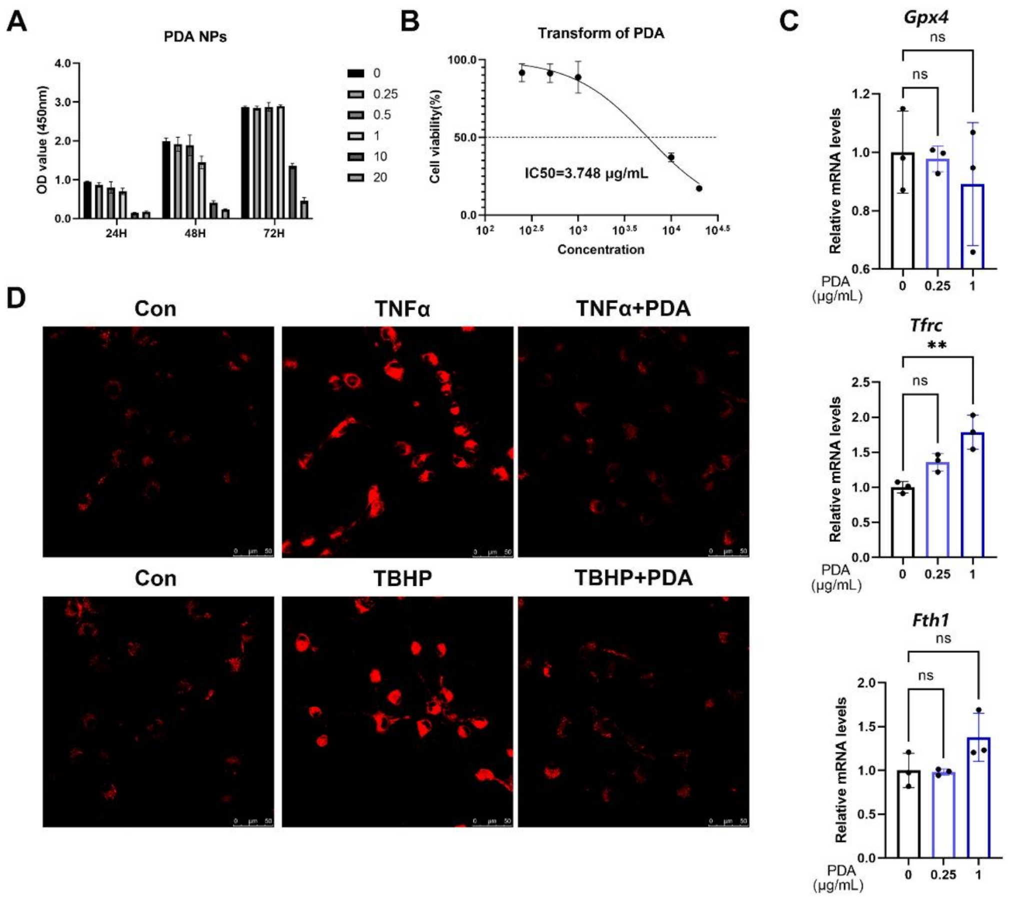

Supplementary Figure 2. (A) Cell Counting Kit-8 assay results of nucleus pulposus (NP) cells stimulated with polydopamine nanoparticles (PDA NPs) at different concentrations (0, 0.25, 0.5, 1, 10, and 20 μg/mL) and periods (ranging from 24-72 h). (B) IC50 assay using CCK-8 in NP cells stimulated with PDA NPs at different concentrations (0, 0.25, 0.5, 1, 10, and 20 μg/mL) for 24 h. (C) Reverse transcription-quantitative PCR analysis was performed to determine the relative mRNA expression levels of *Gpx4*, *Tfrc*, and *Fth1* in NP cells treated with PDA NPs (0, 0.25, and 1 μg/mL) for 24 h; β-actin was the internal reference. (D) Immunofluorescence analysis for ferroOrange of NP cells stimulated with tumor necrosis factor-alpha (TNFα) (10 ng/mL) or tert-butyl hydroperoxide (TBHP) (100 μM) for 12 h and/or pretreated with PDA NPs (1 μg/mL) for 24 h. All data are presented as mean ± standard deviation (SD) from three replicates. $^{*}P<0.05$, $^{**}P<0.01$, $^{***}P<0.001$, and $^{****}P<0.0001$.

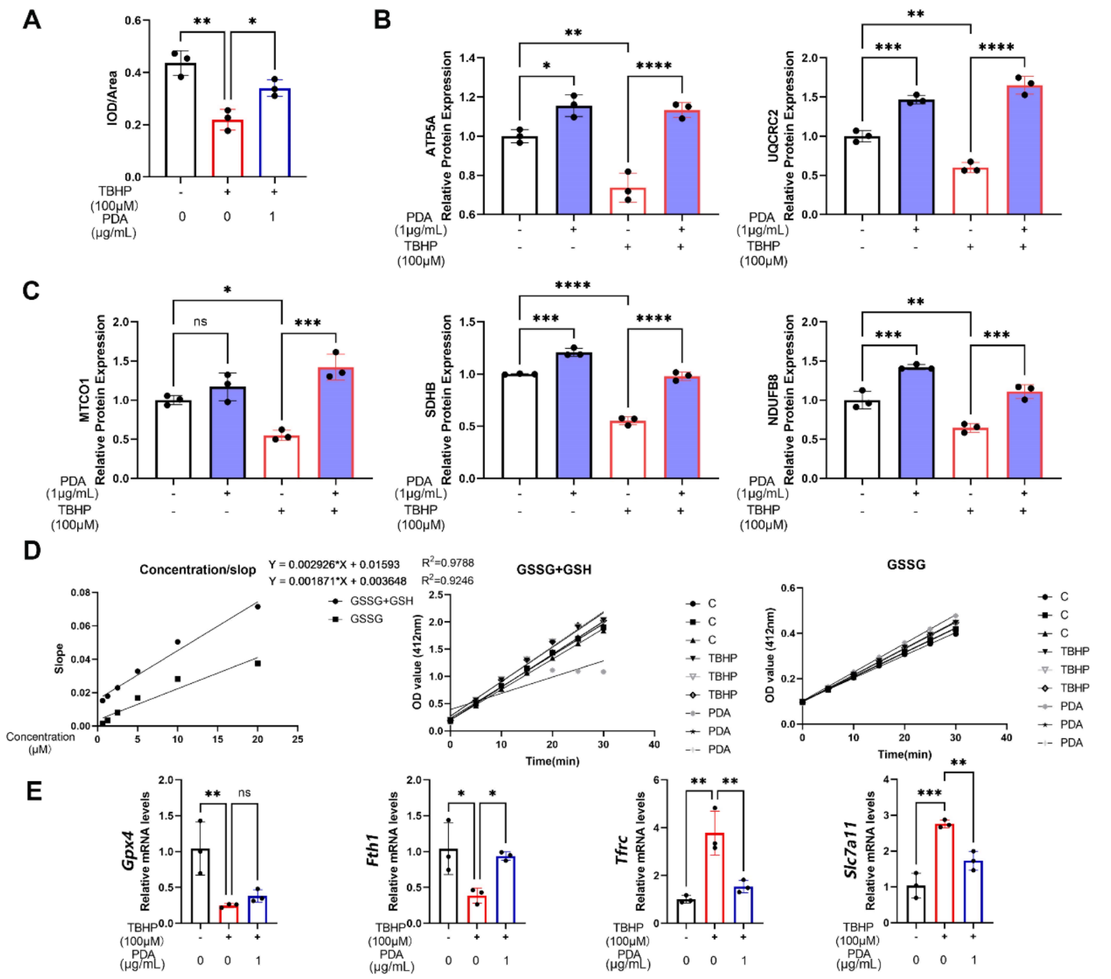

Supplementary Figure 3. (A) Quantification of integrated optical density (IOD)/ 4′,6-diamidino-2-phenylindole (DAPI) staining for nucleus pulposus (NP) cells stimulated with tert-butyl hydroperoxide (TBHP) (100 μM) for 12 h and/or pretreated with polydopamine nanoparticles (PDA NPs) (1 μg/mL) for 24 h. (B, C) Relative protein quantification of the grey scale values for total oxidative phosphorylation (OXPHOS): ATP5A and UQCRC2. (D) Standard curves of glutathione (GSH) and glutathione disulfide (GSSG) slope/concentration, GSSG+GSH/time, and GSSG/time in a GSH assay. (E) Reverse transcription-quantitative PCR analysis for determining the relative mRNA expression levels of *Gpx4*, *Fth1*, *Tfrc*, and *Slc7a11* in NP cells treated with TBHP (100 μM) and/or PDA NPs (1 μg/mL) for 24 h; β-actin was the internal reference. All data are presented as mean ± standard deviation (SD) from three replicates. *P<0.05, **P<0.01, ***P<0.001, and ****P<0.0001.

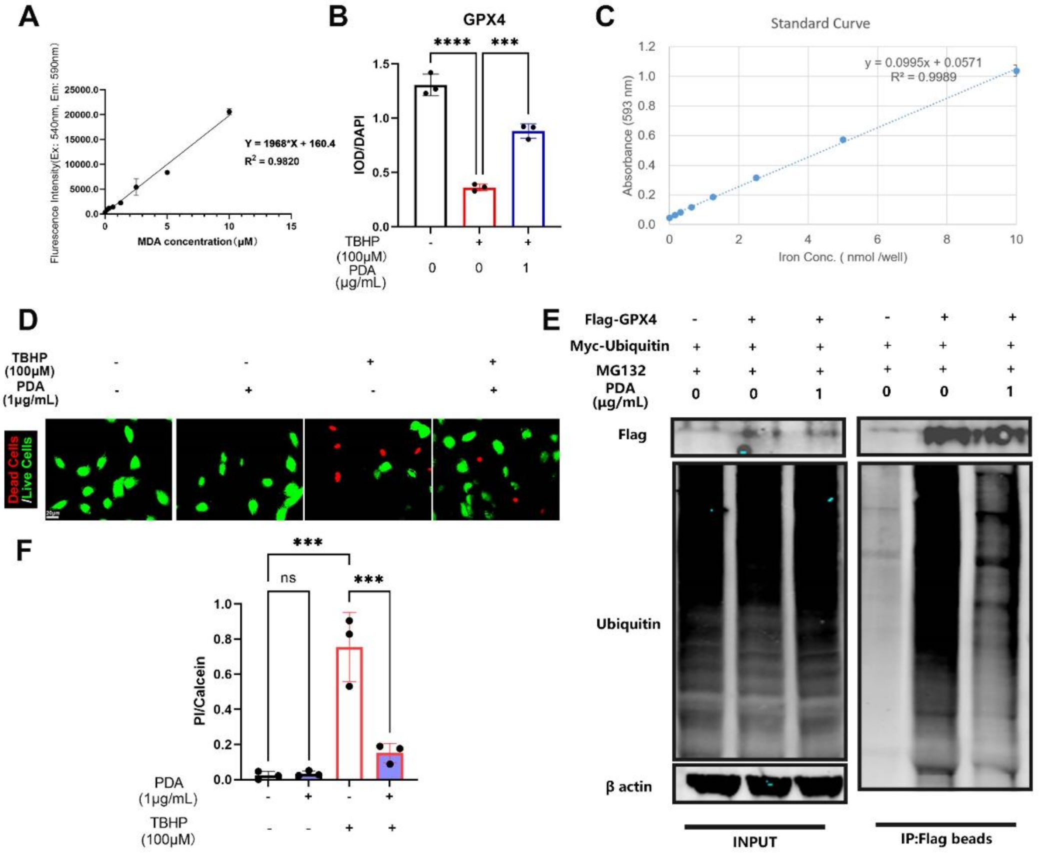

Supplementary Figure 4. (A) Standard curve of malondialdehyde (MDA) concentration in the MDA assay. (B) Quantification of integrated optical density (IOD)/ 4′,6-diamidino-2-phenylindole (DAPI) for GPX4. (C) Standard curve of the iron concentration in the total iron assay. (D) Dead/live cells staining in NP cells stimulated with PDA NPs alone for 24 h, TBHP (100 μM) for 12 h, and/or pretreated with PDA NPs (1 μg/mL) for 24 h. (E) Ubiquitylation analysis of GPX4 in NP cells treated with MG132 (10 μM) and/or PDA NPs (1 μg/mL) using Flag-GPX4 and Myc-ubiquitin plasmids with Flag-tagged beads. (F) Quantification of PI/Calcein staining shown in d. All data are presented as mean ± standard deviation (SD) from three replicates. $^{*}P<0.05$, $^{**}P<0.01$, $^{***}P<0.001$, and $^{****}P<0.0001$.

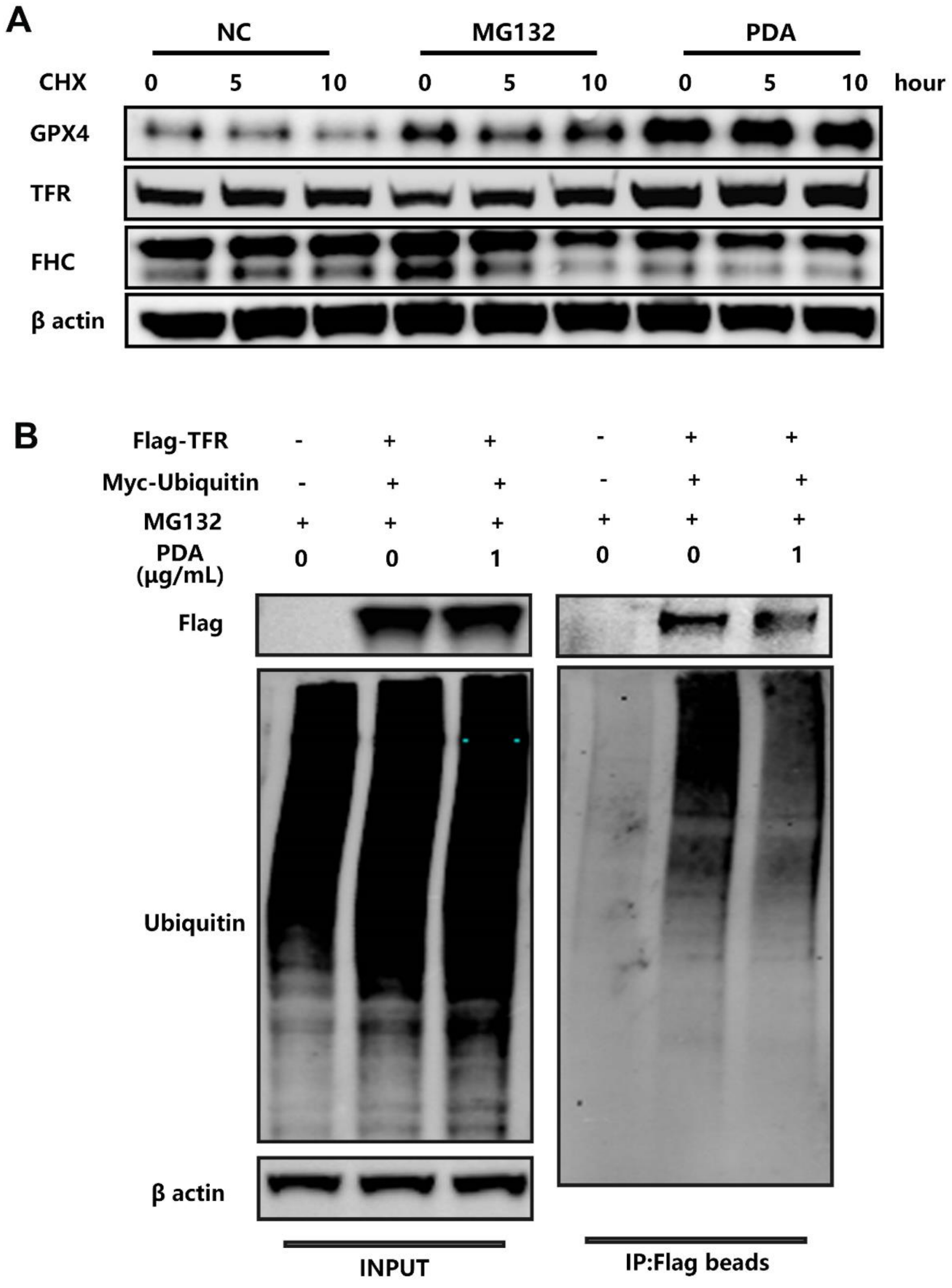

Supplementary Figure 5. (A) Western blot analysis of GPX4, transferrin receptor (TfR), and ferritin heavy chain (FHC) using β-actin as the loading control in nucleus pulposus (NP) cells (pretreated with phosphate buffered saline [PBS], polydopamine nanoparticles [PDA NPs], and MG132) treated with cycloheximide (50 nM) for 0, 5, and 10 h. (B) Ubiquitylation analysis of transferrin receptor (TfR) in 293T cells treated with MG132 (10 μM) and/or PDA NPs (1 μg/mL) using Flag-TFR and Myc-ubiquitin plasmids with Flag-tagged beads.

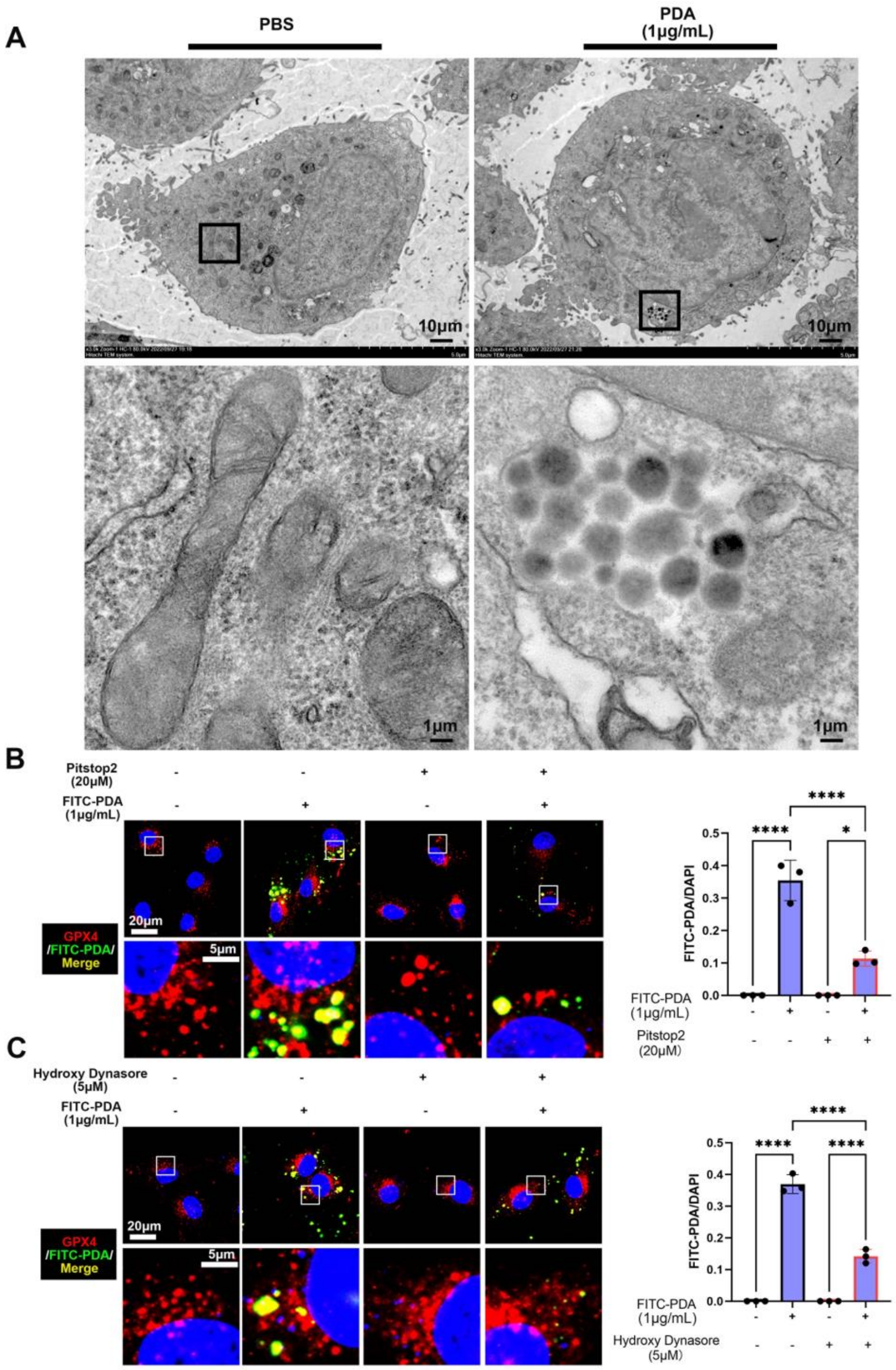

Supplementary Figure 6. (A) Transmission electron microscopy (TEM) analysis of polydopamine nanoparticles (PDA NPs) endocytosis in nucleus pulposus (NP) cells stimulated with PDA NPs 24 h compared to no stimulation. (B) Immunofluorescence and corresponding quantification analysis of Rab5 and FITC-PDA NPs in NP cells stimulated with FITC-PDA NPs alone, Pitstop2 (20 μM), and/or pretreated with FITC-PDA NPs (1 μg/mL) for 24 h. (C)Immunofluorescence and corresponding quantification analysis of Rab5 and FITC-PDA NPs in NP cells stimulated with FITC-PDA NPs alone, Hydroxy Dynasore (5 μM), and/or pretreated with FITC-PDA NPs (1 μg/mL) for 24 h. All data are presented as mean ± standard deviation (SD) from three replicates. $^{*}P<0.05$, $^{**}P<0.01$, $^{***}P<0.001$, and $^{****}P<0.0001$.

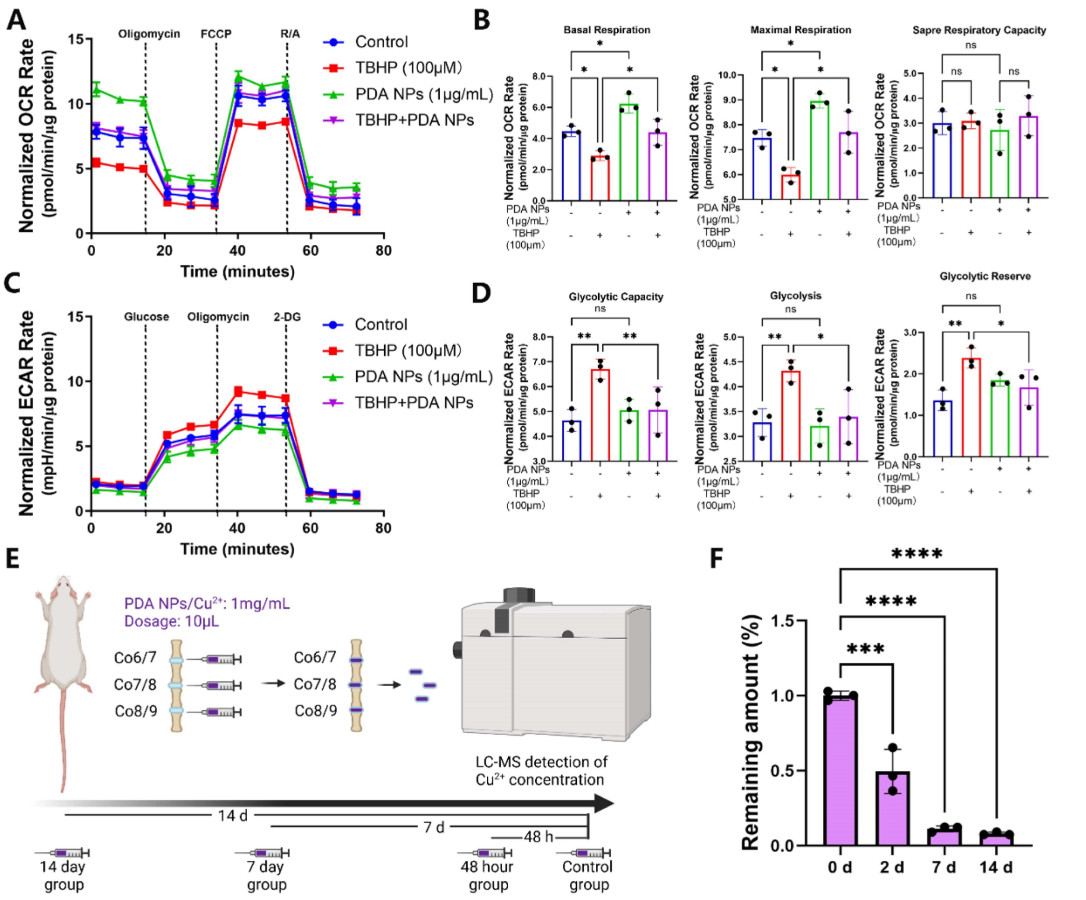

Supplementary Figure 7. (A) Seahorse assay of oxygen consumption rate (OCR) in NP cells stimulated with PDA NPs alone for 24 h, TBHP (100 μM) for 12 h, and/or pretreated with PDA NPs (1 μg/mL) for 24 h. (B) Quantification of basal respiration, maximal respiration, and spare respiratory capacity shown in a. (C) Seahorse assay of extracellular acidification rate (ECAR) in NP cells stimulated with PDA NPs alone for 24 h, TBHP (100 μM) for 12 h, and/or pretreated with PDA NPs (1 μg/mL) for 24 h. (D) Quantification of glycolytic capacity, glycolysis, and glycolytic reserve shown in c. (E) Scheme figure of in vivo PDA NPs degradation assay in rats tails using PDA NPs/ Cu^{2+}.(F) Quantification of Cu^{2+} concentration using ICP-MS detection shown in e. All data are presented as mean ± standard deviation (SD) from three replicates. $^{*}P<0.05$, $^{**}P<0.01$, $^{***}P<0.001$, and $^{****}P<0.0001$.

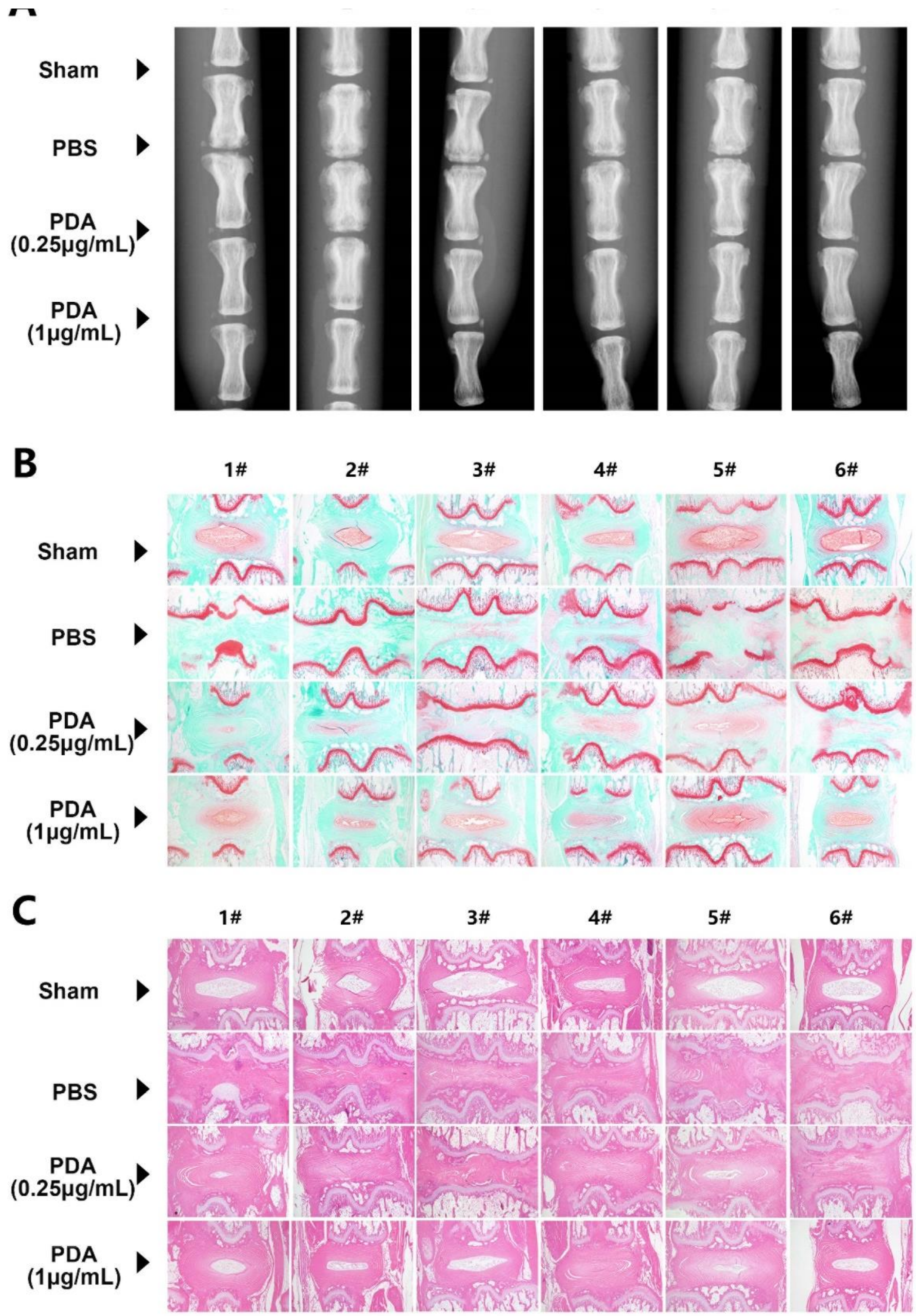

Supplementary Figure 8. (A) Cropped X-ray images of rat tails (n = 6). (B) Safranin O/Fast Green stain of the rat tails (n = 6), focused on the intervertebral discs. (C) Hematoxylin and eosin (H&E) stain of the rat tails (n = 6), focused on the intervertebral discs.

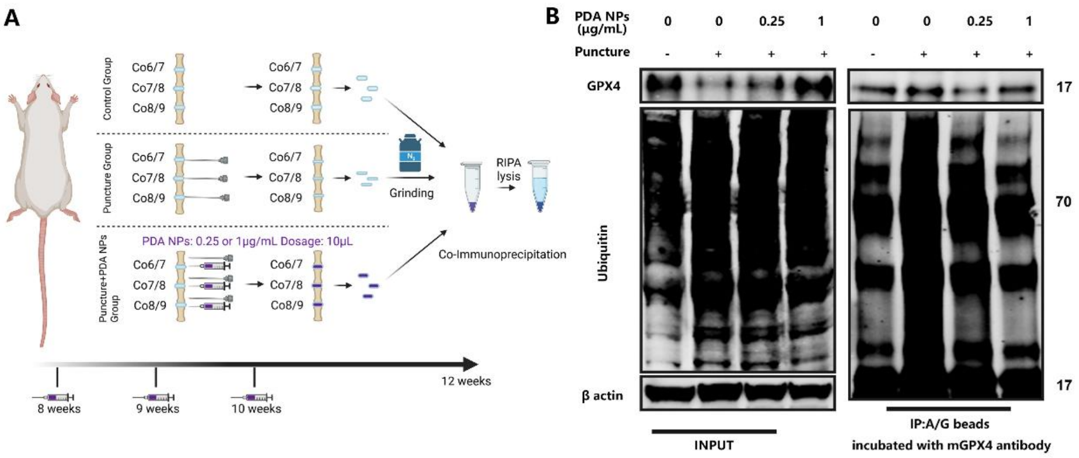

Supplementary Figure 9. (A) Scheme figure of in vivo GPX4 ubiquitylation assay in rat tails divided into four groups (n = 3) using PDA NPs. (B) Ubiquitylation analysis of GPX4 in discs lysate shown in a, the using beads were A/G beads incubated with mouse anti-GPX4 antibody.

Table 1: patient information

Name	*Pfirrmann Grade*	*Gender*	*Age*	*Diagnosis*
Yan	2	female	9	Congenital Spondylolisthesis
Jiang	1	male	12	Congenital Scoliosis
Zhou	1	male	14	Congenital Scoliosis
Ji	1	female	12	Congenital Spondylolisthesis
Shen	4	male	79	Lumbar Spinal Stenosis
Shi	5	female	70	Degenerative Lumbar Spondylolisthesis
Yu	4	female	57	Lumbar Disc Herniation
Gu	5	male	60	Lumbar Disc Herniation

Table 2. Primer information

Gene	Accession Number	Description	5'-Primer-3'
Gpx4	NM_017165.4	F	CCGTCTGAGCCGCTTATTGA
		R	CTGCGAATTCGTGCATGGAG
Fth1	NM_012848.2	F	TGAGCCCTTTGCAACTTCGT
		R	CTTCAGGGCCACATCATCCC
Tfrc	NM_022712.1	F	CCTATATGCTTGGGTAGGAGGC
		R	TACGCGCTTACAATAGCCCA
Slc7a11	NM_001107673.3	F	TCGTCCTTTCAAGGTGCCTC
		R	GGCAGATGGCCAAGGATTTG
β-actin	NM_031144.3	F	GTCCACCCGCGAGTACAAC
		R	GGATGCCTCTCTTGCTCTGG

Table 3. Predicted human phenotypes based on GPX4 by ARCHS.

Rank	Gene Set	Z-score
1	Acute necrotizing encephalopathy (HP:0006965)	4.05284793
2	Abnormal mitochondria in muscle tissue (HP:0008316)	4.02051816
3	Acute encephalopathy (HP:0006846)	3.93202092
4	Myoglobinuria (HP:0002913)	3.83196214
5	Hepatic necrosis (HP:0002605)	3.75065233
6	Hyperglycinuria (HP:0003108)	3.68539062
7	Abnormal activity of mitochondria respiratory chain (HP:0011922)	3.66266894
8	Decreased activity of mitochondria respiratory (HP:0008972)	3.66266894
9	Cerebral edema (HP:0002181)	3.55629574
10	Vertebral compression fractures (HP:0002953)	3.55575021
